# Linking geographic distribution and niche through estimation of niche density

**DOI:** 10.1111/1365-2656.70052

**Published:** 2025-05-08

**Authors:** Tad A. Dallas, Cleber Ten Caten

**Affiliations:** ^1^ Department of Biological Sciences University of South Carolina Columbia South Carolina USA

**Keywords:** common environments, ecological niche, geographic range size, niche density, species distributions

## Abstract

1. The availability of suitable niche space constrains where species can occur geographically. This tie between niche space and geographic space is crucial when estimating species geographic distributions in a changing climate. However, specific combinations of climatic conditions may be overrepresented in geographic space, highlighting the potential disconnect between climatic niche area and geographic range size.
2. We develop a niche density estimator that accounts for the geographic availability of climatic niche space, relate this to traditional estimates of niche area and explore how these niche estimates are related to species geographic range size.
3. To do this, we use data on over 230,000 species recorded in the Global Biodiversity Information Facility, providing a thorough test of the sensitivity of niche estimation technique on geographic range size–climatic niche scaling relationships, and clarifying the link between geographic space and environmental space by considering the density of available environments in environmental space.
4. Niche density was more strongly related to species geographic range size than niche area, highlighting the role of the geographic availability of climatic niche space in biogeographic relationships. As species geographic ranges and environmental conditions change, understanding the ecological and evolutionary determinants of this positive scaling between geographic range size and niche size is an important research frontier.

## Graphical abstract

Species with larger climatic tolerances (niches) may have large or small geographic ranges, dependent on the commonness of favourable climatic conditions across geographic space. Here, we explore the relationship between geographic range size and niche area, while also proposing a novel measure of niche density.

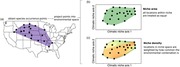

## 1 INTRODUCTION

Species occupy both geographic and environmental space, where these two spaces are implicitly linked through *Hutchinson's duality* (Colwell & Rangel, 2009; Graham et al., 2025; Pulliam, 2000), which acknowledges that the niche is an environmental space that maps onto geographic space, allowing for the reciprocal translation between environmental and geographic projections. The ability of species to specialize on sets of environmental conditions can provide certain advantages, such as the ability to explore those environments more proficiently than other species and potentially be more abundant in those specific conditions (Boulangeat et al., 2012; Maguire Jr., 1973). On the other hand, environmental specialist species can also be more susceptible to changes in the environmental conditions, leading to a higher extinction probability relative to environmental generalist species (Gallagher et al., 2015). Thus, there might be a trade‐off between ecological specialization and species distributions (Clavel et al., 2011; Futuyma & Moreno, 1988), although abundance–occupancy relationships predict the opposite pattern, where generalist species are also more abundant (Gaston, 1999).

But environmental generalism and large geographic range size are not inherently the same thing (Cai et al., 2021; Espeland & Emam, 2011; Graham et al., 2025). Here, environmental generalism would be the ability of a species to persist in a wide range of environments (i.e. have a broad niche), independent of the distribution of population growth rates within niche space (Maguire Jr., 1973). Defining aspects of the species niche (e.g. breadth; Carscadden et al. (2020)) is a central problem in understanding ecological specialization and the corresponding geographic distribution of species (Vela Diaz et al., 2020; Verberk et al., 2010). Specialist and generalist species are usually thought to have narrow and broad niche breadths, respectively (Boulangeat et al., 2012; Clavel et al., 2011), where evidence suggests that species with large geographic ranges tend to also have broader niche breadth (Bozinovic et al., 2011; Dallas & Kramer, 2022; Kambach et al., 2019; Slatyer et al., 2013). The projection of species geographic distributions into environmental space (*e*‐space) allows the quantification of niche (or something resembling a niche) from species occurrence data. However, measuring ecological specialization of a species is a challenging task given the multidimensional and the multi‐scale nature of the niche (Colwell & Futuyma, 1971). Finally, methodological choices can affect these niche estimates (Cano‐Barbacil et al., 2022) which can further complicate the assessment of these relationships.

Estimating aspects of the niche has led to a variety of terms and characterization approaches (Dolédec et al., 2000; Hurlbert, 1978; Smith, 1982). The most common measures tend to be *niche breadth* and *niche position* (sometimes referred to as *marginality*; Cano‐Barbacil et al., 2022; Carscadden et al., 2020). Niche breadth attempts to quantify the *span* of the niche, or the range of environmental conditions that a species may occupy. Niche position attempts to estimate the distance between the environmental space where a species is found relative to some background on the available environments. While niche breadth estimates the range of environments a species is likely to persist in, niche position estimates the species *use* of the environments relative to the environments available. The key difference is that niche position considers the geographic distribution, or at least the geographic commonness and rarity, of environmental conditions, while niche breadth does not. However, most analyses do not necessarily consider the actual density of environments available, or they constrain environmental density by the set of sampled sites when calculating these niche measures (Dolédec et al., 2000; Vela Diaz et al., 2020). That is, the commonness of certain environments, and the resulting projection of geographic space into environmental space, is inherently constrained by the sampled area or set of sampled sites (Cano‐Barbacil et al., 2022).

This highlights a fundamental issue. How do we define niche position or breadth in a manner which considers the density of environmental conditions in a given geographic extent? Further, what are the effects of the density of environmental conditions on our measurement of the niche? Previous definitions of niche similarity based on environmental density have focused on the overlap between species in environmental niche space, only considering the environments occupied by a species, or more frequently an entire sampled community (Brown & Carnaval, 2019; Fridley et al., 2007). Further, models which use pseudo‐absence (or *background*) data effectively explore the density of environmental conditions where a species occurs relative to the density of that environment across geographic space (Broennimann et al., 2012; Drake et al., 2006). These efforts have been incredibly useful to push niche overlap to consider the density of environmental space, but they do not address estimation of niche area or density relative to the geographic distribution of available environmental space. Most species that have small niches often also have constrained niche density (Brown & Carnaval, 2019), but some might specialize in commonly observed environmental conditions (which they may or may not occupy) and have large niche density despite having small niches. Further, estimates of niche position that are conditional upon the sampled community are useful when the community is the unit of study, but perhaps less so when we want to explore species‐level patterns independently (e.g. estimates of niche position will be sensitive to the geographic distribution of sampled sites).

The decision of how to estimate niche position is non‐trivial in many ways. Constraining the estimates of niche position by the sampled sites leads to a potentially false description of the range and distribution of environmental conditions available to a species. Defining niche position independent of the sampling process can help alleviate some of these issues, and start to get at the potential of a species to expand to novel geographic locations within the species environmental tolerances. Here, we explore how considering the density of available environments influences the estimation of niche position, and how this can influence established scaling patterns such as the relationship between geographic range size and climatic niche area or position. To do this, we use data on over 230,000 species recorded in the Global Biodiversity Information Facility to explore how niche estimation technique influences the relationship between geographic range size and the climatic niche. Further, we design a measure of niche density which considers the availability of different environments in geographic space. Together, we provide evidence that *specialist* species may specialize in widely distributed and common environments, and explore how accounting for this influences the relationship between geographic range size and climatic niche area.

## 2 METHODS

### 2.1 The global biodiversity information facility

The Global Biodiversity Information Facility (GBIF) provides a platform to aggregate species occurrence data in a standardized data format, with over 2 billion species occurrence records. The GBIF data may be accessed programmatically in at least two ways in the *R* programming environment; querying records using rgbif (Chamberlain & Boettiger, 2017) or gbifdb (Boettiger, 2021). We use gbifdb, which offers snapshots of the entire GBIF database through the integration of Parquet with *R*. We used the release of GBIF from April 2024 accessible from gbifdb, consisting of over 2 billion total occurrence records.

For each species, we used CoordinateCleaner (Zizka et al., 2019) to remove occurrence points in capital cities, country centroids and at large research institutions, as well as duplicated occurrences, those that have country information where the occurrence is not in the country, and those occurrence points located at 0 latitude and 0 longitude. We only considered species with greater than 25 occurrence records in our analyses after cleaning the data as described above. This resulted in 235,426 species. We removed taxonomic classes corresponding to fish species (Myxini, Petromyzontida, Hyperoartia, Chondrichthyes, Actinopterygii and Sarcopterygii), as aquatic points close enough to land area may provide estimates of niche area that we did not wish to include. We also removed *Homo sapiens* and several domesticated species (*Canis familiaris*, *Ovis aries*, *Bos taurus*, *Capra hircus*, *Felis catus*, *Cavia porcellus*, *Equus asinus*, *Bubalus bubalis*, *Camelus dromedarius*, *Apis mellifera*, *Equus caballus* and any species whose latin name ended in ‘domesticus’ or ‘domestica’). This resulted in a total of 233,681 species spanning 1073 orders and 5854 families. The most common orders corresponded to butterflies and moths (Lepidoptera; 17,860 species), beetles (Coleoptera; 15,747 species) and angiosperms (Asterales; 10,376 species). These data are extensively used for species distribution modelling and biogeographic research, but are not without limitation. For instance, sampling and detection bias are naturally present, some records may lead to a mismatch in time between the climate layers and the occurrence records, and a portion of records coming from iNaturalist (approximately 8% of GBIF currently) have altered latitude and longitude coordinates for rare or endangered species, which can bias estimates of geographic range size and niche area (Contreras‐Díaz et al., 2023). We explore this in the Supplement by breaking down relationships based on IUCN red list threat category. GBIF remains a vital resource for explorations at the scale of our analyses. Finally, we recognize that this is not the full extent of the GBIF database, but due to computational constraints and our threshold of at least 25 observations, we do not consider the full set of over 1 million species.

### 2.2 Estimation of geographic range

Many methods have been developed to estimate species geographic range and climatic niche area (Burgman & Fox, 2003; Lichti & Swihart, 2011; Quinn et al., 1996). Each method makes assumptions about the structure of the climatic niche or the spatial distribution of a species across a landscape. For sake of simplicity, we use the convex hull of all sampled points (after removal of potentially spurious occurrences as described above). The minimum convex polygon is defined as the smallest area that connects all occurrence points with no interior angles. As such, the method does not utilize occurrence points from the interior of the geographic range but uses the extreme points to define the limits of occurrence for a species. We mask species geographic ranges by the environmental data raster, meaning that geographic range area only considers the land area, which is important for species that occur in multiple continents with large bodies of water in between. More restrictive approaches attempt to reduce the weight of occurrences that are geographically removed from the rest (e.g. alpha hulls), but these approaches require further parameterization and may still be prone to sampling and detection biases (Darroch & Saupe, 2018). How the geographic range should be defined is still very much an open question (Pappalardo et al., 2020; Sheth et al., 2012), though previous explorations of the relationship between geographic range area and niche area found qualitatively similar results across multiple range estimators (Dallas & Kramer, 2022). We explore this further in the Supplemental Materials.

### 2.3 Estimation of the climatic niche

We operationalize the species niche as the set of climatic space a species occupies for a given time and space, commonly referred to as the realized niche (Soberon, 2007). To be clear, estimating the niche from species occurrence data is implicitly flawed in the context of the Hutchinsonian niche, as the niche is a persistence threshold, not an occurrence threshold. That is, occurrence points may represent viable populations that are capable of persisting, or could be sink populations or transients (geographic locations that would not allow for persistence). Without information on species demographic parameters and population densities, we are constrained to treat occurrence points as geographic locations which allow for species persistence. The climatic niche space was defined by using a reduced environmental space. We used a set of 56 environmental variables from WorldClim (Hijmans et al., 2005) at 2.5 arc‐degree resolution. The WorldClim variables (*n* = 37), containing elevation and monthly information on minimum and maximum temperature and precipitation, and the BioClim variables (*n* = 19), containing derived quantities such as temperature seasonality and mean annual precipitation, are well‐tested and well‐used climatic data (Barbet‐Massin & Jetz, 2014), showing high degree of similarity with other geospatial data sources such as Chelsa (Karger et al., 2017). The high‐dimensional environmental space was transformed into a low‐dimensional space through principal components analysis, in which the first two axes explained over 77% of the total global climatic variation (Kambach et al., 2019; Kriticos et al., 2014). We created a two‐dimensional space with the first two axes, consisting of a 0.05 arc‐degree resolution raster of 851,200 cells encompassing the full range of environmental space. Each cell in the environmental raster corresponds to some small range of potential values in terms of the two environmental PCA axes, and the value within the cell corresponds to the number of geographic cells which correspond to that particular environment. We explore how the resolution of this raster influences niche density estimates in the supplement, finding that niche density estimates are unaffected by raster resolution.

### 2.4 Climatic niche density estimation

**FIGURE 1:**
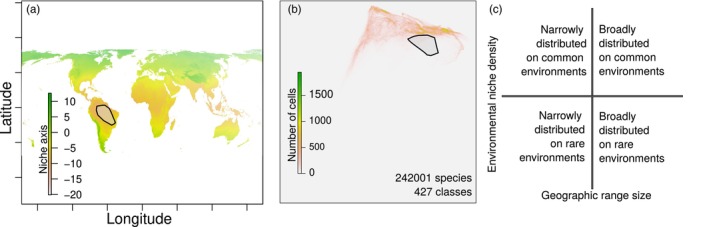
Species geographic occurrence records based on species distributions (a) may cover different portions of the available niche space (b). The global environmental space in terms of temperature and precipitation monthly averages was compressed to two axes (*a* shows the first axis, *b* shows both). Polygons in geographic (a) and niche (b) space link species distributions with the niche in a way that highlights the role of common environments. This suggests that small‐ranged species do not necessarily have small niches, and large‐ranged species may specialize in common environments (c).

The lower dimensional representation of the global environmental space allows us to estimate the niche by projecting the range of environments a species was found to occur, and then delineating the minimum convex polygon in environmental space, similar to how we estimate geographic range area above. The area of this polygon is a standard way to estimate species niche area (Warren et al., 2010), but it does not take into consideration the commonness or rarity of those environmental conditions. That is, if we consider two climatic niche axes, the total environmental space is a plane, even though some environmental conditions within that space are much more represented than others in geographic space. To address this, we treated the environmental space as a raster under an equal area projection, where each cell contained the number of raster cells from the geographic space in which that set of environmental conditions occurred (Figure 1). The result is a three‐dimensional surface, as now we include the additional frequency layer within the environmental space. To estimate niche density, we use the same minimum convex polygon as described above, but now sum the values falling within the polygon, serving to characterize the niche not only as the range of climatic space occupied but also by the commonness of those climatic conditions in geographic space. The resulting estimate corresponds to the potential geographic area (in terms of number of cells in the raster) that the species could potentially occupy. This inherently links niche density to geographic range size, with disconnects between niche density and geographic range providing information on the relative utilization of potential habitat by the species (i.e. niche filling; Moore et al., 2023).

### 2.5 Niche density and geographic range size

Niche density putatively estimates the potential geographic area available to a species given its environmental niche. However, there may be disconnects between estimates of geographic range size and estimates of niche density, driven either by species underfilling their potential geographic range size or overestimating species geographic ranges, leading to areas within the geographic range that may actually be unsuitable based on niche estimation. Importantly, estimates of geographic range size assume that all the area interior of the polygon is suitable for species. This assumption may create a situation where geographic range size is larger than our estimate of niche density, as niche density estimates the total geographic area corresponding to environmental conditions within the species niche, while there may be unsampled areas of environmental space within the geographic range polygon which the species may occupy that would not be part of the niche density estimate.

We recognize that using the global climatic space may be extreme, as species that are geographically constrained may occupy common climatic conditions which are wholly unreachable to them. However, the underlying point of estimating the niche in this manner is to explore the *potential* of the species to occupy larger areas, and the disconnect between the realized niche we observe and the true fundamental niche a species could occupy is important. We do note that we are still not estimating the true fundamental niche, but by considering the global environmental space, we are including those sites that the species could potentially occur at without considering the constraints of dispersal to the given location and the role of biotic interactions in allowing species persistence. Researchers have discussed the implications of considering accessible area when training species distribution models (Barve et al., 2011; Soberón & Osorio‐Olvera, 2023), as dispersal limitation may provide restrictions on the scaling between climatic niche area and geographic range size (Colwell & Rangel, 2009; Soberón & Osorio‐Olvera, 2023). In fact, a commonly used measure of niche separation only uses the environmental conditions corresponding to the sampled sites, making it an incredibly local measure that may be strongly influenced by the sampling design (Dolédec et al., 2000). Here, we use the global climatic space, but we explore the influence of available land area in the Supplemental Materials by constraining the geographic and environmental space by only considering species occurring in the Americas, with quite similar overall findings. We encourage researchers to use a geographic extent that best addresses their research question.

### 2.6 Comparison of niche area and niche density

**FIGURE 2:**
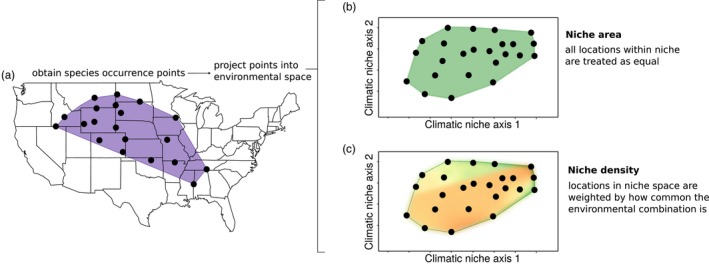
The proposed estimate of niche density considers the commonness and rarity of the environmental conditions in geographic space. From species occurrence records in geographic space (a), we project these into a two‐dimensional environmental space to estimate niche area (b) and niche density (c), which capture different aspects of the niche. Niche density accounts for the availability of environments in geographic space.

We explored the agreement between estimates of niche area and niche density for our set of 234,478 species using a Spearman's rank correlation to account for any potential non‐linearities in the relationship between these two measures of the niche. We further explore how defining the niche using the more traditional niche area and our measure, which considers the niche space as a density surface, by exploring the relationship between geographic range size and the climatic niche. This relationship has been claimed to be quite general, though it is often fairly weak and influenced by geography and species traits (Dallas & Kramer, 2022; Slatyer et al., 2013). One potential reason for the weak relationship could be that species may specialize in small regions of niche space that are very common in geographic space, leading to disconnects between estimates of niche area and the reality of potential geographic range size. An overview of our approach and a comparison of niche area and niche density is described in Figure 2.

### 2.7 The null expectation of geographic range size and niche area/density

Due to spatial autocorrelation in environmental conditions, it is expected that there will be a positive relationship between geographic range size and niche area. In our calculation of density, we count all geographic cells which correspond to environments within the species niche, likely leading to an even stronger expected relationship between geography and niche. This is informative, as the disconnect between our estimate of niche density and geographic range size can be used to explore niche and geographic range filling (Moore et al., 2023). We explore the potential null relationship between geographic range size and niche area/density by simulating 100,000 virtual species distributed randomly across the landscape. We select initial terrestrial points at random, incorporating geographic range size variation by sampling nearby occurrence points based on a normal distribution with standard deviation between 1° and 70° latitude or longitude. We sampled 20, 100 and 500 occurrence points to explore the effects of the number of observations. Geographic range size and niche area/density measures were calculated as described above. There were some simulated species for which it was not possible to estimate niche area or niche density, resulting in slightly different numbers of species across simulations of 20 (*n* = 75,194), 100 (*n* = 88,203) and 500 (*n* = 92,527) sampled occurrence points.

## 3 RESULTS

### 3.1 Comparison of niche area and niche density approaches

**FIGURE 3:**
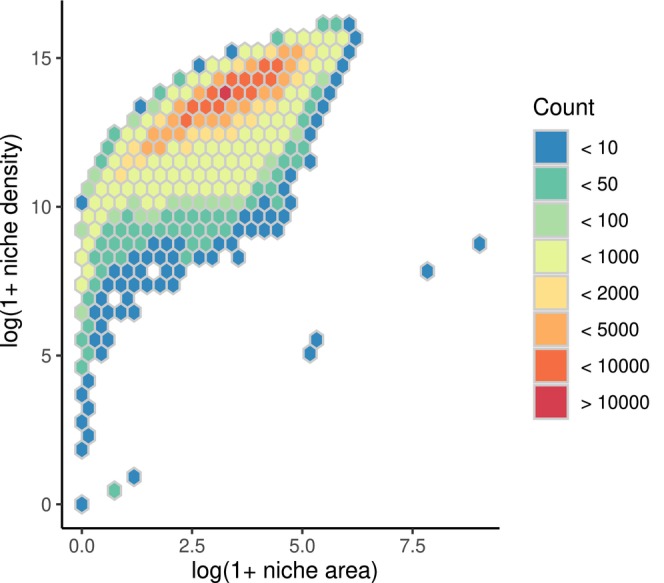
Niche area—defined as the area of the minimum convex polygon in niche space—was positively related to niche density, which we defined as the sum of the geographic cells that contain environmental conditions within the minimum convex polygon that is the species niche. Species with a small niche area may occupy common environments, leading to a quicker increase in niche density estimates with increasing niche area. However, this saturates as niche area includes all environmental space, weighting extreme and rare conditions equivalent to common environmental conditions, reflected as the saturating response as niche area increases. Cell colour refers to the number of species within that bin.

We found a positive relationship between niche area and niche density (Figure 3; *ρ* = 0.80, *p* < 0.0001), suggesting that species with large niche areas (those that occupy a large portion of niche space), also tend to occupy more common environments (i.e. have larger niche density). However, the relationship was markedly non‐linear, with niche density saturating as niche area values increased (Figure 3). This suggests that niche density increases fairly quickly with niche area (i.e. small regions of environmental space may still contain a high density of environmental conditions if those conditions are common). The saturating relationship is due to niche area being bounded by the total environmental space, while those extreme edge conditions in environmental space do not contribute much to niche density (as these extreme environments tend to be quite rare).

### 3.2 Geographic range size and climatic niche area relationships

**FIGURE 4:**
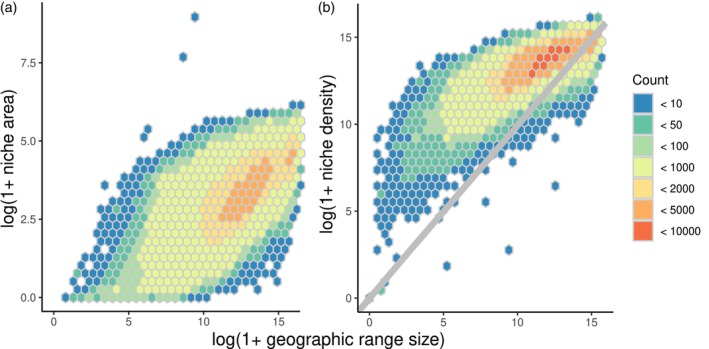
Geographic range size was positively related to both niche area (a) and niche density (b). The stronger relationship observed for niche density is potentially a result of niche density integrating the density of the niche space, weighted by the geographic commonness of that set of environmental conditions. Cell colour refers to the number of species within that bin. The grey line in panel b corresponds to the 1:1 relationship between estimated geographic range size and niche density, where values above the line correspond to potential range underfilling and values below the line correspond to potential overestimation of geographic range size.

Both niche area (*ρ* = 0.66, *p* < 0.0001) and niche density (*ρ* = 0.78, *p* < 0.0001) were related to geographic range size (Figure 4). The influence of niche density can be observed in the relative tightness of the bounds of the relationship relative to using niche area measures, which suggests that niche area is a measure which reflects the range of environmental conditions a species occupies independent of the commonness of those environmental conditions in geographic space. By incorporating information on the commonness of environmental conditions, niche density estimates provide a tighter link with species geographic range. This is most notable for species with small geographic ranges (Figure 4), which have quite low niche area measures, but higher niche density values (i.e. smaller range species are specializing on relatively common environmental conditions). Differences between geographic range size and niche density were observed (Figure 4), with species with smaller geographic ranges tending to underfill their potential geographic range given their niche, and species with larger geographic range actually having larger ranges than their niche density. This likely suggests that there are environments within the geographic range for which we did not have species occurrence data, so these environments were in the geographic range but not in the niche density estimates. This could also occur if geographic range size was overestimated as a result of outlier occurrence points affecting range area estimates.

Based on a Moran's *I* at the family level, we found a phylogenetic signal, with more closely related families tending to have similar geographic range sizes (*o* = 0.001, *e* = −0.0002, *p* < 0.0001), niche densities (*o* = 0.0006, *e* = −0.0002, *p* < 0.0001) and niche areas (*o* = 0.001, *e* = − 0.0002, *p* < 0.0001). We use a phylogenetic least squares regression to account for taxonomic relationships at the family level in the Supplemental Materials. However, due to the constraints on building such a large phylogeny and having to simplify to family‐level taxonomic relationships, the analysis was performed using mean values for niche area and density. When accounting for taxonomic relationships, we still observe qualitatively similar results to our correlation analyses. We also explored how geographic extent influenced these results by limiting the species considered to only those found in the Americas, finding similar results (see Supporting Information). Constraining the area examined is more likely to influence niche density estimates, as the global environmental density may be quite different from the environmental density of the Americas, while niche area estimates do not incorporate information on geographic distribution of environments. Still, the current approach of only considering the environmental space of sampled sites is far more restrictive than either approach.

### 3.3 The null expectation of geographic range size and niche area/density

Given the constraints of our null model simulations, we see a weaker relationship between geographic range size and either niche area or niche density, at least relative to the empirical data. That is, there was still positive scaling between geographic range size and the niche, but there were far more simulated species with fairly large geographic ranges and small niche areas or densities than for the empirical species (see Supporting Information).

## 4 DISCUSSION

We found support for the relationship between geographic range area and niche area, but this relationship was far from predictive, as species with moderately sized geographic ranges could have a wide range of niche areas. This is because niche area assumes that all environmental conditions within the niche can be treated equally. By considering the commonness and rarity of environmental conditions, we clearly show that niche density is more strongly related to geographic range area. Our results highlight the role of species with narrow environmental preferences to common habitats, which is reflected in niche density, but not niche area. We further highlight the utility of niche density as the potential geographic range area given the estimated niche, highlighting that many species with smaller geographic ranges are likely underfilling their potential geographic range size. Together, we developed an approach for estimating niche density which allows clearer linkages between niche space and geographic space, highlighting the utility in understanding geographic range filling (Moore et al., 2023) and disconnects caused by attempting to infer habitat specialism from geographic range size.

Niche density measures can complement previous approaches at quantifying aspects of the niche, including niche breadth and position, where breadth is traditionally measured as the range of climatic variable(s) a species can occur at and position relates the mean climatic condition for a species to some background data on available environments. These measures are incredibly useful to characterize the niche (Carscadden et al., 2020), but are admittedly somewhat coarse approximations. Niche density combines two important features of each of the measures, by using information on the climatic tolerance ranges across multiple axes (similar to niche breadth) and by considering the background density of available environmental space (similar to niche position). By leveraging the distribution of common and rare climatic conditions in geographic space, we can start to disentangle habitat specialism and geographic range size (where specializing on a common habitat can result in a relatively small niche size but relatively large geographic range size). In doing so, we find a clearer relationship between niche and geographic range size than was estimated using traditional niche area estimates.

While niche density estimates can provide information on the distribution of common and rare climatic environments, it does not mean that a species specializing in common climatic environments will inherently be more widespread. Dispersal limitation, biotic interactions and many other factors will determine where a species can occur geographically (Godsoe et al., 2017; Soberón & Osorio‐Olvera, 2023). Further, the use of occurrence data to define niche boundaries is not without issues, as transient or non‐persisting populations may still be included in estimates of the niche, and occurrence data may span a temporal range that is not represented by the climatic layers used to define the niche. If many species were specializing in common climatic conditions (or if species were generalists on fairly rare climatic conditions), we would expect that niche density would not necessarily be as strongly related to geographic size. However, by calculating niche density as the sum of geographic space encompassed by the species climatic tolerances, we may have allowed for aspects of geographic range size to inform our estimates of the niche, meaning that the relationship is clearer due to the inherent scaling relationship with larger geographic ranges corresponding to larger niche areas (and densities). This is a problem for nearly all attempts to relate the niche and geographic range size (Colwell & Rangel, 2009; Pulliam, 2000; Slatyer et al., 2013).

The importance of the density of environmental conditions cannot be overstated. It is the reason why many species distribution modelling approaches sample *pseudo‐absence* or *background* points, as these points represent the distribution of the relevant niche axes (Soberón & Nakamura, 2009). Previous efforts to apply these types of niche concepts in biogeographic studies have yielded approaches which incorporate niche density (Dolédec et al., 2000), but which constrain the niche density surface to the set of sampled sites. By considering the global (or regional, see Supplement) niche density surface, we explore the inherent link between niche and geographic space (Pulliam, 2000) to understand the distribution of species. This approach could be further used to explore niche overlap between species, with the goal of disentangling geographic overlap from niche overlap, or understanding niche partitioning and evolution (Sexton et al., 2017). Further, this approach could be extended or allow for corrections, such as the weighting of niche density by climatic suitability estimated from a species distribution model or by species occurrence density (Broennimann et al., 2012). Previous approaches estimating the niche as a response surface (Maguire Jr., 1973) have assumed that demographic performance is enhanced within the niche interior (Martínez‐Meyer et al., 2013). Our measure of niche density does not assume this, but could be extended to capture the potential differential contribution of different environments to species demographic rates, potentially by weighting common and rare environments based on species estimated demographic rates or corresponding niche position.

Considering niche density is especially important considering the role of climate change on shifting species geographic distributions (Hellmann et al., 2012) and potentially leading to niche evolution (Quintero & Wiens, 2013; Tingley et al., 2009). Niche density estimates may reflect the underlying spatial effects of a changing climate in a way that niche area cannot. To affect niche area, the species would have to be found in an environment outside of the current climatic range, while niche density acknowledges that climate change will alter the availability of niche space across geographic gradients. That is, niche area will only change when the species range of climatic conditions a species persists in is altered, but niche density estimates will change simply as a function of the availability of climatic conditions changing. This has the potential to inform how species may respond to climatic shifts, by explicitly considering the shifting distribution of the geographic availability of climatic niche axes.

## AUTHOR CONTRIBUTIONS

All authors contributed to the original idea and final manuscript writing. TAD performed the analyses.

## FUNDING INFORMATION

This work has been performed with funding to Tad Dallas from the National Science Foundation (NSF‐DEB‐2017826 and NSF‐DEB‐2420769).

## CONFLICT OF INTEREST STATEMENT

The authors have no conflicts of interest to declare.

## Supporting information


**Figure S1.** Niche area—defined as the area of the minimum convex polygon in niche space—was positively related to niche density, which we defined as the sum of the geographic cells which contain environmental conditions within the minimum convex polygon that is the species niche.
**Figure S2.** Constraining the species considered and environmental niche space to only the Americas resulted in findings qualitatively similar to the main text.
**Figure S3.** Given the set of null species simulations, we see a weak positive relationship between geographic range size and niche area.
**Figure S4.** Given the set of null species simulations, we see a weak positive relationship between geographic range size and niche density.
**Figure S5.** Geographic range size estimation using minimum convex polygons (x‐axis) compared to estimates from alpha hulls across a range of parameterizations of *α*.
**Figure S6.** Correlations between geographic range size estimates (right) and niche density estimates (left) at different levels of data thresholding (either 5% or 10% extreme points removed from the geographic range).
**Figure S7.** The relationship between geographic range size and climatic niche density was not strongly affected by the removal of extreme geographic values prior to estimation of geographic range size and climatic niche density for the 500 randomly sampled species explored.
**Figure S8.** Niche area – defined as the area of the minimum convex polygon in niche space – was positively related to niche density, which we defined as the sum of the geographic cells which contain environmental conditions within the minimum convex polygon that is the species niche.
**Figure S9.** Geographic range size was positively related to niche density, regardless of IUCN threat status.
**Figure S10.** The fraction of records per species considered in our analyses which came from iNaturalist observations.
**Table S1.** Pearson's correlations between both geographic range size (as estimated using minimum convex polygon) and niche area and corresponding alpha hull estimates along a gradient of *α* values.
**Table S2.** Phylogenetic least squares regression models on the relationship between niche area and niche density as a function of geographic range size.

## Data Availability

All data are publicly available. *R* code to acquire and analyse data is available on figshare at https://doi.org/10.6084/m9.figshare.24158340.
