# Supplementary material for: Linking geographic distribution and niche through estimation of niche density
Source: J Anim Ecol. 2025 May 8;94(6):1221–30. doi: 10.1111/1365-2656.70052 (PMC12134432; doi:10.1111/1365-2656.70052)
Supplement: Supplementary file 1 — Figure S1. Niche area—defined as the area of the minimum convex polygon in niche space—was positively related to niche density, which we defined as the sum of the geographic cells which contain environmental conditions within the minimum convex polygon that is the species niche. Figure S2. Constraining the species considered and environmental niche space to only the Americas resulted in findings qualitatively similar to the main text. Figure S3. Given the set of null species simulations, we see a weak positive relationship between geographic range size and niche area. Figure S4. Given the set of null species simulations, we see a weak positive relationship between geographic range size and niche density. Figure S5. Geographic range size estimation using minimum convex polygons (x‐axis) compared to estimates from alpha hulls across a range of parameterizations of α. Figure S6. Correlations between geographic range size estimates (right) and niche density estimates (left) at different levels of data thresholding (either 5% or 10% extreme points removed from the geographic range). Figure S7. The relationship between geographic range size and climatic niche density was not strongly affected by the removal of extreme geographic values prior to estimation of geographic range size and climatic niche density for the 500 randomly sampled species explored. Figure S8. Niche area – defined as the area of the minimum convex polygon in niche space – was positively related to niche density, which we defined as the sum of the geographic cells which contain environmental conditions within the minimum convex polygon that is the species niche. Figure S9. Geographic range size was positively related to niche density, regardless of IUCN threat status. Figure S10. The fraction of records per species considered in our analyses which came from iNaturalist observations. Table S1. Pearson's correlations between both geographic range size (as estimated using minimum convex polygon) and niche [file JANE-94-1221-s001.zip › supplement.pdf]

Supplemental Material

Linking geographic distribution and niche through estimation of niche density

Tad Dallas and Cleber Ten Caten

Constraining the geographic space to the Americas

Here, we constrain the geographic area examined to only the Americas, separated out by considering only those points that were bounded longitudinally between 54 and 105.3. This does include some islands, but no aquatic cells were counted towards the geographic range estimations. By limiting the area explored, we do reduce the number of species with available data from 234,523 species in the main text to 18,091 species in the Americas. Despite this reduction in species numbers, we see qualitatively similar results for the scaling of geographic range size and both niche area and niche density (Figure S1 and S2).

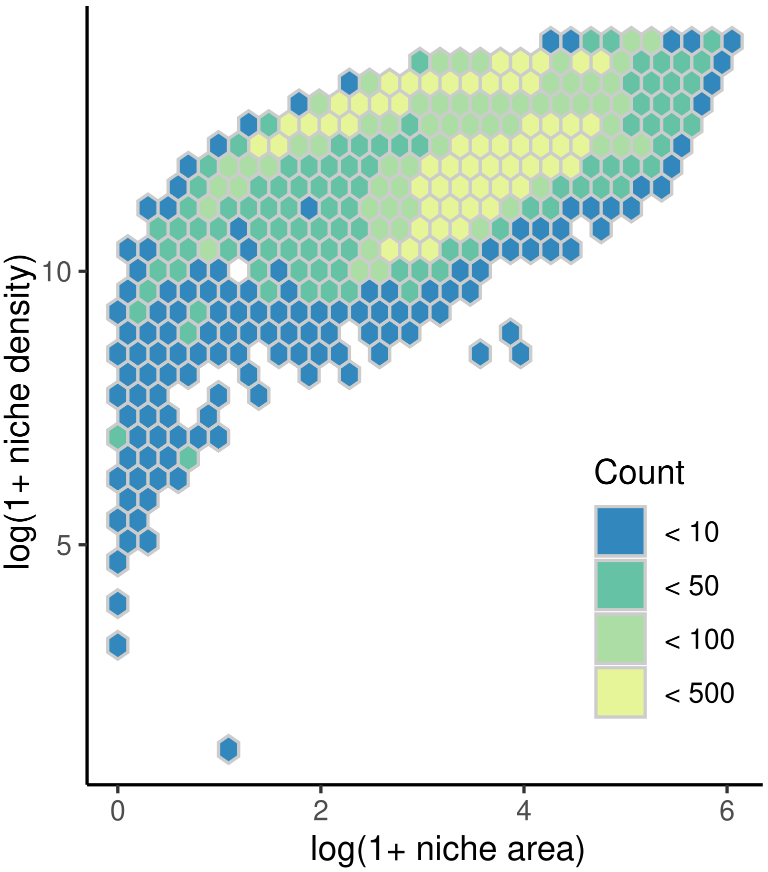

Figure S1: Niche area – defined as the area of the minimum convex polygon in niche space – was positively related to niche density, which we defined as the sum of the geographic cells which contain environmental conditions within the minimum convex polygon that is the species niche. Species with small niche area may occupy common environments, leading to a quicker increase in niche density estimates with increasing niche area. However, this saturates as niche area includes all environmental space, weighting extreme and rare conditions equivalent to common environmental conditions, reflected as the saturating response as niche area increases.

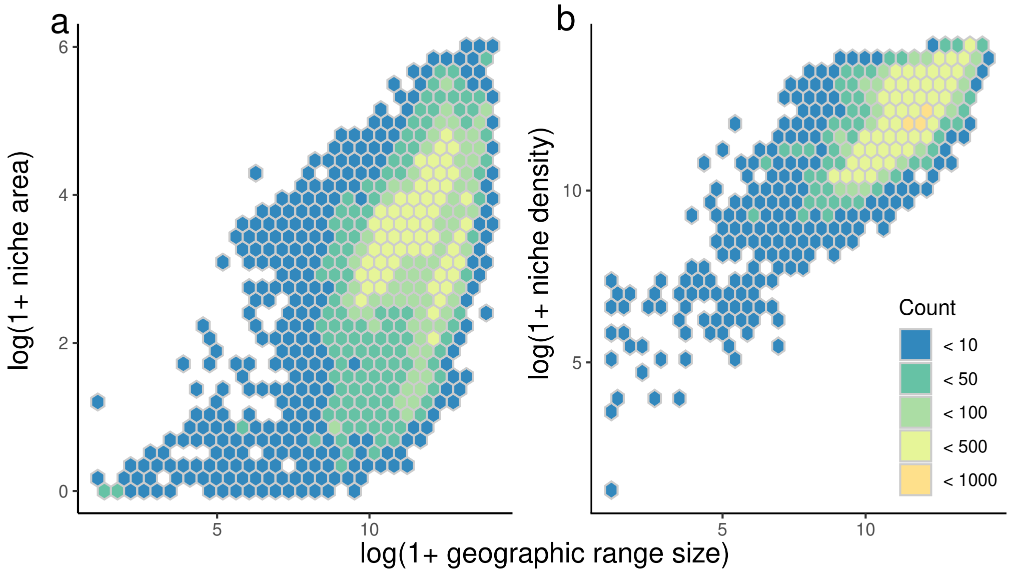

Figure S2: Constraining the species considered and environmental niche space to only the Americas resulted in findings qualitatively similar to the main text. Geographic range size was positively related to both niche area (a) and niche density (b). The stronger relationship observed for niche density is potentially a result of niche density integrating the density of the niche space, weighted by the geographic commonness of that set of environmental conditions.

Evaluating a null model of niche – geographic range size relationships

If geographic ranges were placed randomly on a map, it might be expected that there would be a positive relationship between geographic range size and niche area or density (Slatyer *et al.*, 2013). Here, we explore the underlying null relationship expected given species distributions on a landscape with inherent (but varying degrees of) spatial autocorrelation in environmental niche space. To do this, we select 100,000 terrestrial points, and sample a fixed number of points (20, 100, or 500) within a latitudinal and longitudinal range of each point (between 1 and 70 degrees latitude and longitude). This provides a range of values of geographic range size, and bypasses the influence of latitudinal richness gradients and other factors by placing the initial 100,000 points randomly across the globe.

The number of occurrence points used (20, 100, or 500) did not strongly influence the null relationships observed between geographic range size and either niche area (Figure S3) or niche density (Figure S4). Further, both null relationships appeared more variable in the null model simulations relative to the empirical data. This could simply be a function of how we simulated the null species, though we did manage to successfully capture the range of geographic range sizes and niche area/density values observed in the empirical data. Spatial structure in where species occur (e.g., the latitudinal diversity gradient) may bias species occurrences to certain geographic areas, which could explain part of the difference between null expectation and our observed relationships. In particular, the null species simulations for niche density appear to create a bimodal distribution of null niche density estimates (Figure S4), suggesting that species with the same geographic range size could have markedly different niche densities. This would potentially be driven by the initial random point, as species distributed in rare environments could reduce niche density for the same given geographic area.

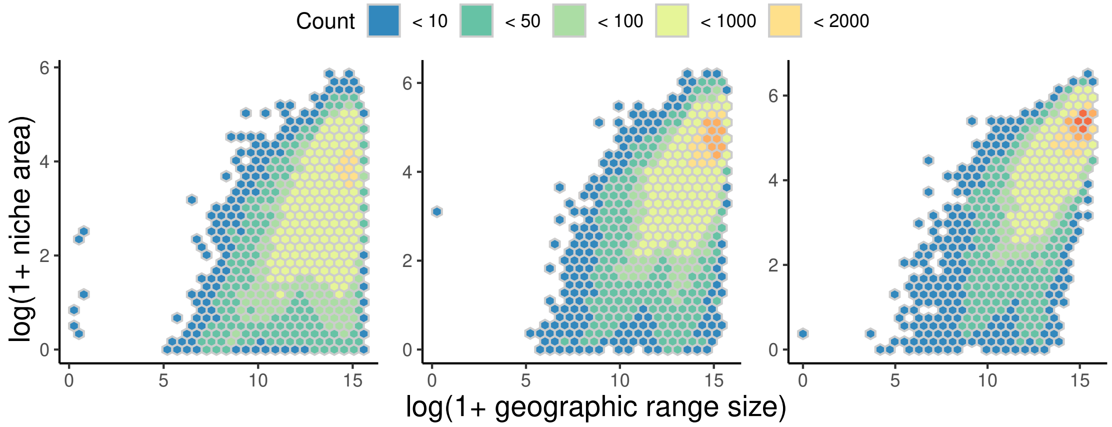

Figure S3: Given the set of null species simulations, we see a weak positive relationship between geographic range size and niche area. Colored cells correspond to the number of species with that given range of geographic range size and niche area estimates. Panels correspond to the number of occurrence points used to simulate the null species (20, 100, or 500 from left to right).

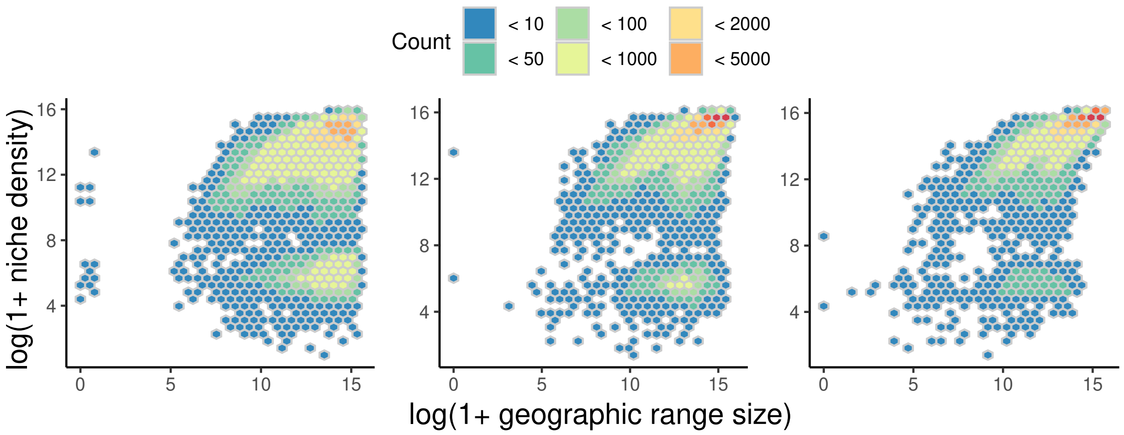

Figure S4: Given the set of null species simulations, we see a weak positive relationship between geographic range size and niche density. Colored cells correspond to the number of species with that given range of geographic range size and niche density estimates. Panels correspond to the number of occurrence points used to simulate the null species (20, 100, or 500 from left to right).

The use of alpha hulls compared to convex hulls

We explored how different approaches to estimating geographic range size might influence our overall findings. To do this, we estimated geographic range size using alpha hulls with 4 different parameterizations for the α value (0.1, 1, 10, 100). The estimation of alpha hulls was computationally demanding and sensitive to seemingly random segfaults. Due to these issues, we only calculated alpha hulls for the first 7500 species, only 3964 of which had sufficient data to estimate species geographic range size and niche area.

As α becomes large, the estimates of geographic range size more closely resemble the minimum convex polygon (Figure S5). We also explored how related our alpha hull estimates were to our minimum convex polygons, finding significant correlations except for one parameterization of α ($\alpha = 0.1$). Further, geographic range size as estimated using alpha hulls was still related to niche area across all parameterizations of α (Table S1). However, the smallest α value considered is potentially too small, resulting in range estimates of less than 1 km^2 when minimum convex polygons are over 10000 km^2.

Table S1: Pearson's correlations between both geographic range size (as estimated using minimum convex polygon) and niche area and corresponding alpha hull estimates along a gradient of α values. Alpha hull estimates were extremely variable, and different parameterizations of α lead to even alpha hull estimates themselves that were uncorrelated ($\alpha = 0.1$ was unrelated to α=100).

Variable	α	t	ρ	p-value
Geographic area	0.1	0.311	0.0049	0.7557
	1	8.821	0.1388	< 0.0001
	10	20.927	0.3154	< 0.0001
	100	78.528	0.7802	< 0.0001
Niche area	0.1	4.489	0.0711	< 0.0001
	1	21.767	0.3267	< 0.0001
	10	39.785	0.5342	< 0.0001
	100	35.847	0.4948	< 0.0001

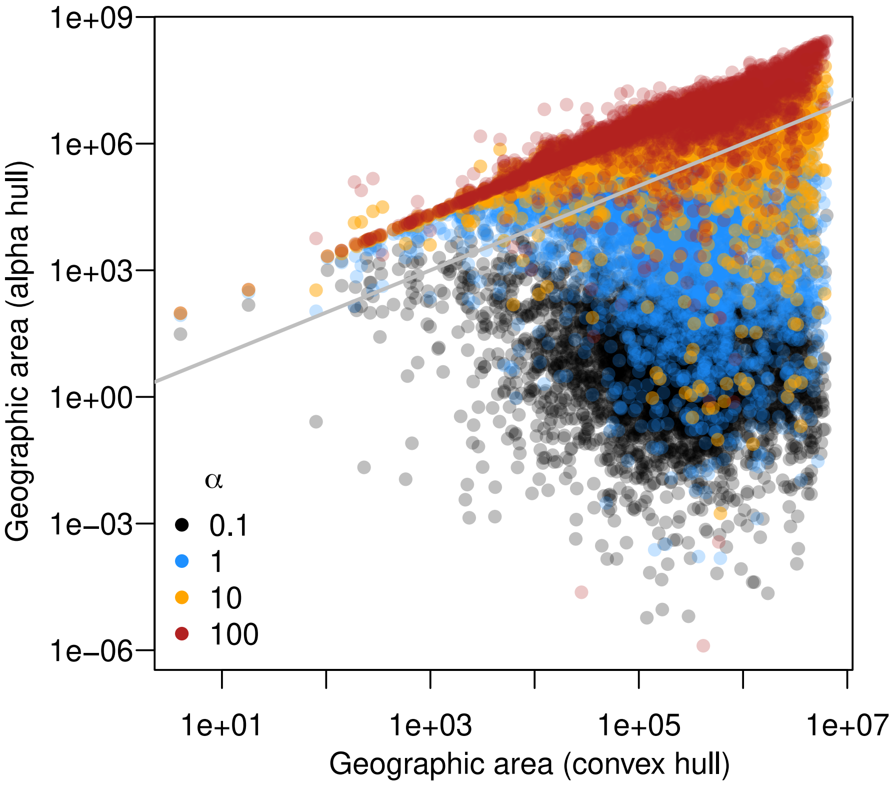

Figure S5: Geographic range size estimation using minimum convex polygons (x-axis) compared to estimates from alpha hulls across a range of parameterizations of α. Defining the value of α is just as subjective as defining which points to consider when estimating a species geographic range. For some parameterizations of α (e.g., $\alpha = 0.1$), we observe no relationship between geographic range estimation techniques for this set of 3964 species.

Removing extreme points does not influence geographic range and niche density

Minimum convex polygons may be sensitive to extreme values, potentially overinflating estimates of geographic range size and subsequent estimates of climatic niche density. We explored this above by considering alpha hulls, finding them difficult to parameterize and oddly easy to segfault a machine. We explore another option for estimating species geographic ranges and niche density here, which is to remove some percentage of the most extreme values. To do this, we first delineate a convex hull, then calculate the distance of all occurrences to that convex hull, removing the top $X\%$ of the points (where X is either 5% or 10% of the sampled points) with the smallest distance to the hull. It was infeasible to do this analysis for all species considered in the main text, so we randomly sampled 500 species to explore this question. The resulting estimates of geographic range size and climatic niche density were highly correlated (Figure S6) and did not strongly influence the resulting relationship between geographic range size and climatic niche density (Figure S7).

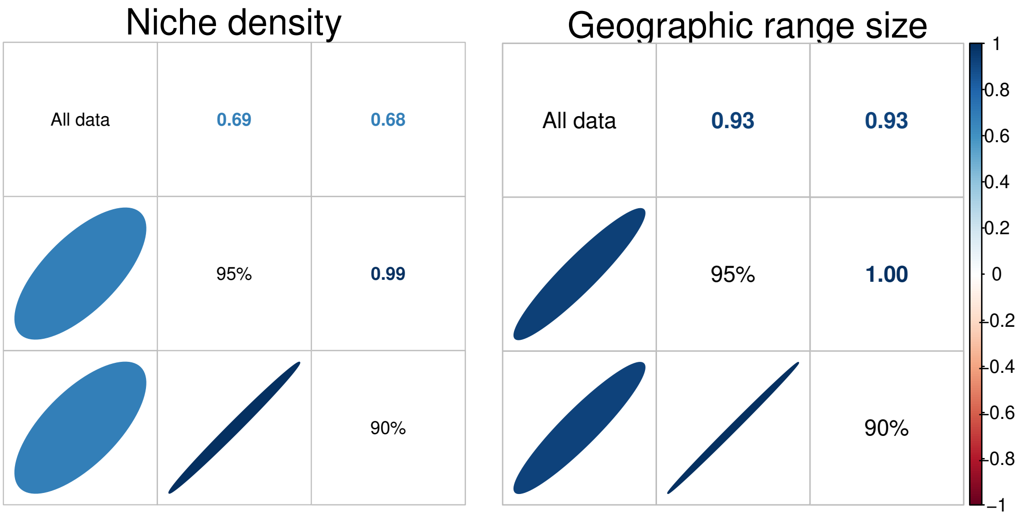

Figure S6: Correlations between geographic range size estimates (right) and niche density estimates (left) at different levels of data thresholding (either 5% or 10% extreme points removed from the geographic range). Linear models forced through the origin between non-thresholded (y) and thresholded (x) data indicate slopes that are close to 1 for geographic range size (β=1.032 and β=1.039 for 5% and 10% respectively) and niche density (β=1.244 and β=1.265 for 5% and 10% respectively).

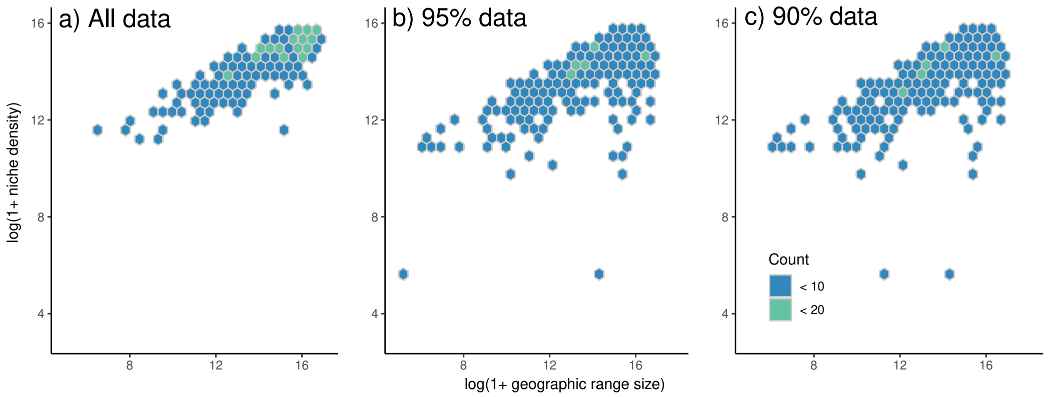

Figure S7: The relationship between geographic range size and climatic niche density was not strongly affected by the removal of extreme geographic values prior to estimation of geographic range size and climatic niche density for the 500 randomly sampled species explored. We either kept all occurrence points (a), 95% (b), or 90% (c) for the estimation of species geographic range, still using minimum convex polygon to delineate ranges.

Accounting for taxonomic relationships among species

Niche area, niche density, and geographic range size contained a taxonomic signal at the family level (results reported in main text). To explore if this influenced the resulting relationships between these variables, we used phylogenetic least squares regression on mean values of niche density, niche area, and geographic range size at family level. We found qualitatively similar results (Table S2), supporting our findings about the scaling between niche area, niche density, and geographic range size.

Table S2: Phylogenetic least squares regression models on the relationship between niche area and niche density as a function of geographic range size. The slope coefficient estimates for geographic range size in both models are similarly positive in the correlation analyses in the main text performed at species-level, while these models were based on family-level averages.

Model	Variable	Estimate	SE	t	p-value
Niche area	(Intercept)	-0.733	0.045	-16.186	<0.0001
	geography	0.335	0.004	90.387	<0.0001
Niche density	(Intercept)	-2.229	0.212	-10.517	<0.0001
	geography	0.517	0.017	29.831	<0.0001

Exploring the influence of IUCN threat status

Threatened or endangered species may deviate from expected geographic range size–climatic niche area relationships as a result of their declining populations, possible endemicity to smaller subsets of geographic or climatic niche space, or due to biases in geographic coordinates from GBIF caused by the slight jittering of locations to preserve a measure of anonymity by iNaturalist (Contreras-Díaz *et al.*, 2023). Here, we break down the relationship between niche area and niche density into the more common IUCN threat categories (data deficient, least concern, near threatened, vulnerable, endangered, and critically endangered). Many species did not have a clear match to the GBIF species names, and their IUCN threat status was classified as 'unknown'. We found similar scaling relationships between niche area and niche density (Figure S8), as well as similar relationships between niche density and geographic range size (Figure S9). Finally, we also report the fraction of records which came from iNaturalist for each species considered in the analysis (Figure S10).

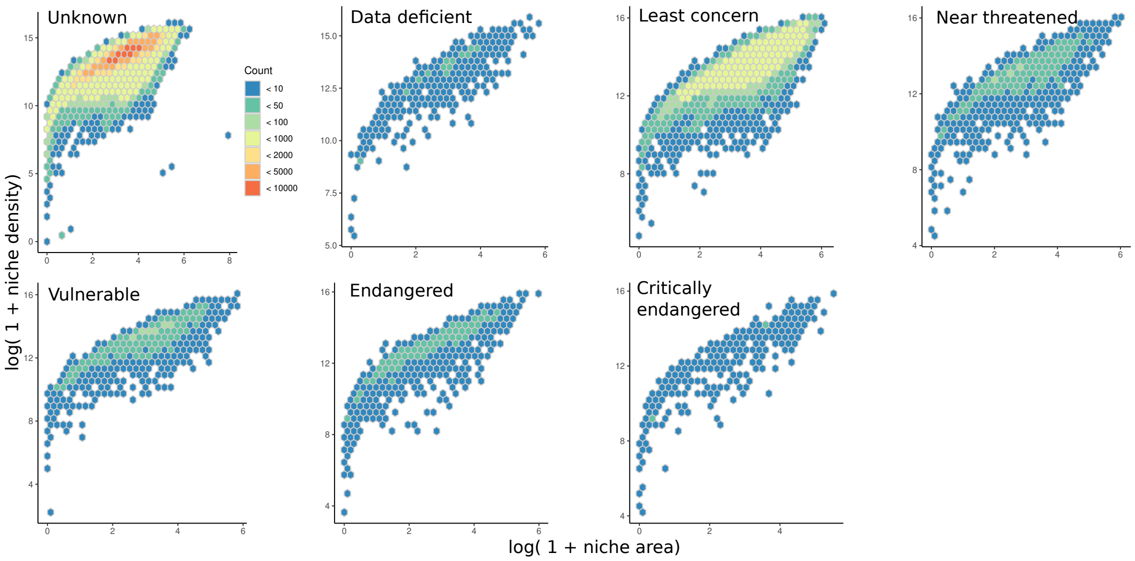

Figure S8: Niche area – defined as the area of the minimum convex polygon in niche space – was positively related to niche density, which we defined as the sum of the geographic cells which contain environmental conditions within the minimum convex polygon that is the species niche. Species with small niche area may occupy common environments, leading to a quicker increase in niche density estimates with increasing niche area. However, this saturates as niche area includes all environmental space, weighting extreme and rare conditions equivalent to common environmental conditions, reflected as the saturating response as niche area increases. Cell color refers to the number of species within that bin.

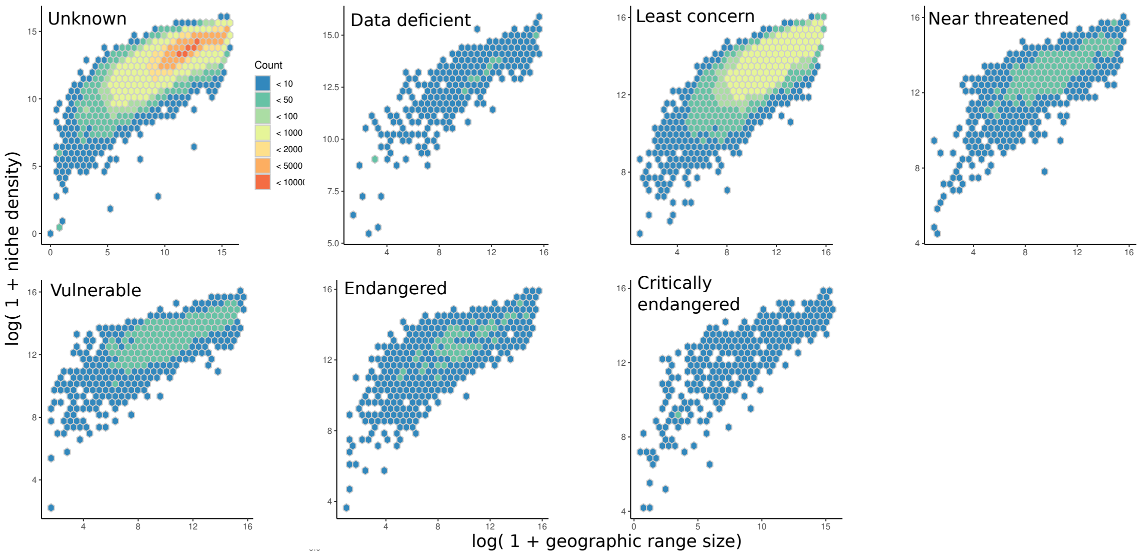

Figure S9: Geographic range size was positively related to niche density, regardless of IUCN threat status. Cell color refers to the number of species within that bin.

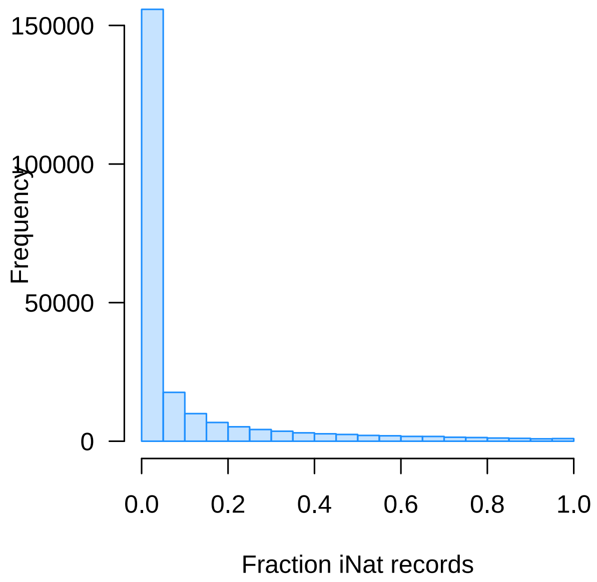

Figure S10: The fraction of records per species considered in our analyses which came from iNaturalist observations.
