# Supplementary figures and images for: Linking geographic distribution and niche through estimation of niche density
Source: J Anim Ecol. 2025 May 8;94(6):1221–30. doi: 10.1111/1365-2656.70052 (PMC12134432; doi:10.1111/1365-2656.70052)

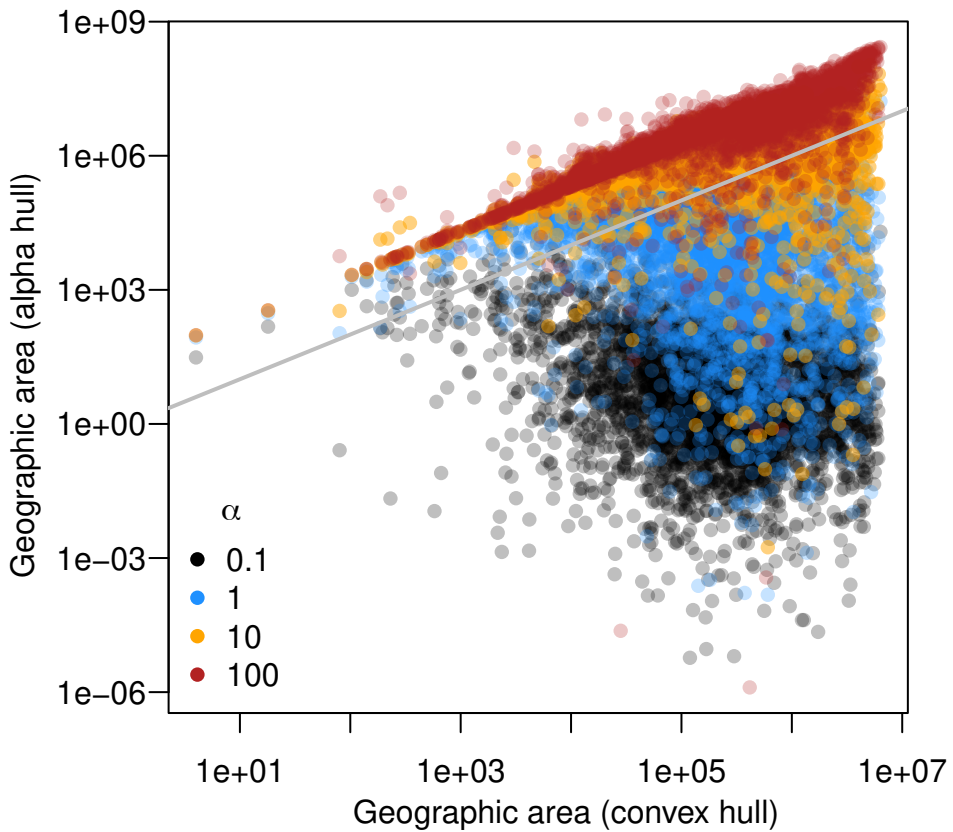

Supplement: Supplementary file 1 — Figure S1. Niche area—defined as the area of the minimum convex polygon in niche space—was positively related to niche density, which we defined as the sum of the geographic cells which contain environmental conditions within the minimum convex polygon that is the species niche. Figure S2. Constraining the species considered and environmental niche space to only the Americas resulted in findings qualitatively similar to the main text. Figure S3. Given the set of null species simulations, we see a weak positive relationship between geographic range size and niche area. Figure S4. Given the set of null species simulations, we see a weak positive relationship between geographic range size and niche density. Figure S5. Geographic range size estimation using minimum convex polygons (x‐axis) compared to estimates from alpha hulls across a range of parameterizations of α. Figure S6. Correlations between geographic range size estimates (right) and niche density estimates (left) at different levels of data thresholding (either 5% or 10% extreme points removed from the geographic range). Figure S7. The relationship between geographic range size and climatic niche density was not strongly affected by the removal of extreme geographic values prior to estimation of geographic range size and climatic niche density for the 500 randomly sampled species explored. Figure S8. Niche area – defined as the area of the minimum convex polygon in niche space – was positively related to niche density, which we defined as the sum of the geographic cells which contain environmental conditions within the minimum convex polygon that is the species niche. Figure S9. Geographic range size was positively related to niche density, regardless of IUCN threat status. Figure S10. The fraction of records per species considered in our analyses which came from iNaturalist observations. Table S1. Pearson's correlations between both geographic range size (as estimated using minimum convex polygon) and niche [file JANE-94-1221-s001.zip › alphaHull.pdf]

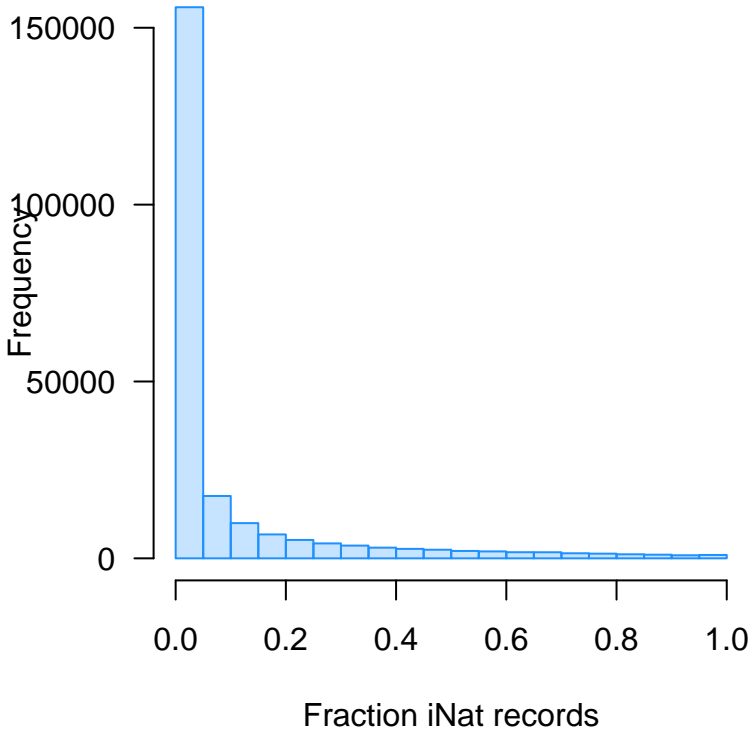

Supplement: Supplementary file 1 — Figure S1. Niche area—defined as the area of the minimum convex polygon in niche space—was positively related to niche density, which we defined as the sum of the geographic cells which contain environmental conditions within the minimum convex polygon that is the species niche. Figure S2. Constraining the species considered and environmental niche space to only the Americas resulted in findings qualitatively similar to the main text. Figure S3. Given the set of null species simulations, we see a weak positive relationship between geographic range size and niche area. Figure S4. Given the set of null species simulations, we see a weak positive relationship between geographic range size and niche density. Figure S5. Geographic range size estimation using minimum convex polygons (x‐axis) compared to estimates from alpha hulls across a range of parameterizations of α. Figure S6. Correlations between geographic range size estimates (right) and niche density estimates (left) at different levels of data thresholding (either 5% or 10% extreme points removed from the geographic range). Figure S7. The relationship between geographic range size and climatic niche density was not strongly affected by the removal of extreme geographic values prior to estimation of geographic range size and climatic niche density for the 500 randomly sampled species explored. Figure S8. Niche area – defined as the area of the minimum convex polygon in niche space – was positively related to niche density, which we defined as the sum of the geographic cells which contain environmental conditions within the minimum convex polygon that is the species niche. Figure S9. Geographic range size was positively related to niche density, regardless of IUCN threat status. Figure S10. The fraction of records per species considered in our analyses which came from iNaturalist observations. Table S1. Pearson's correlations between both geographic range size (as estimated using minimum convex polygon) and niche [file JANE-94-1221-s001.zip › inat.pdf]

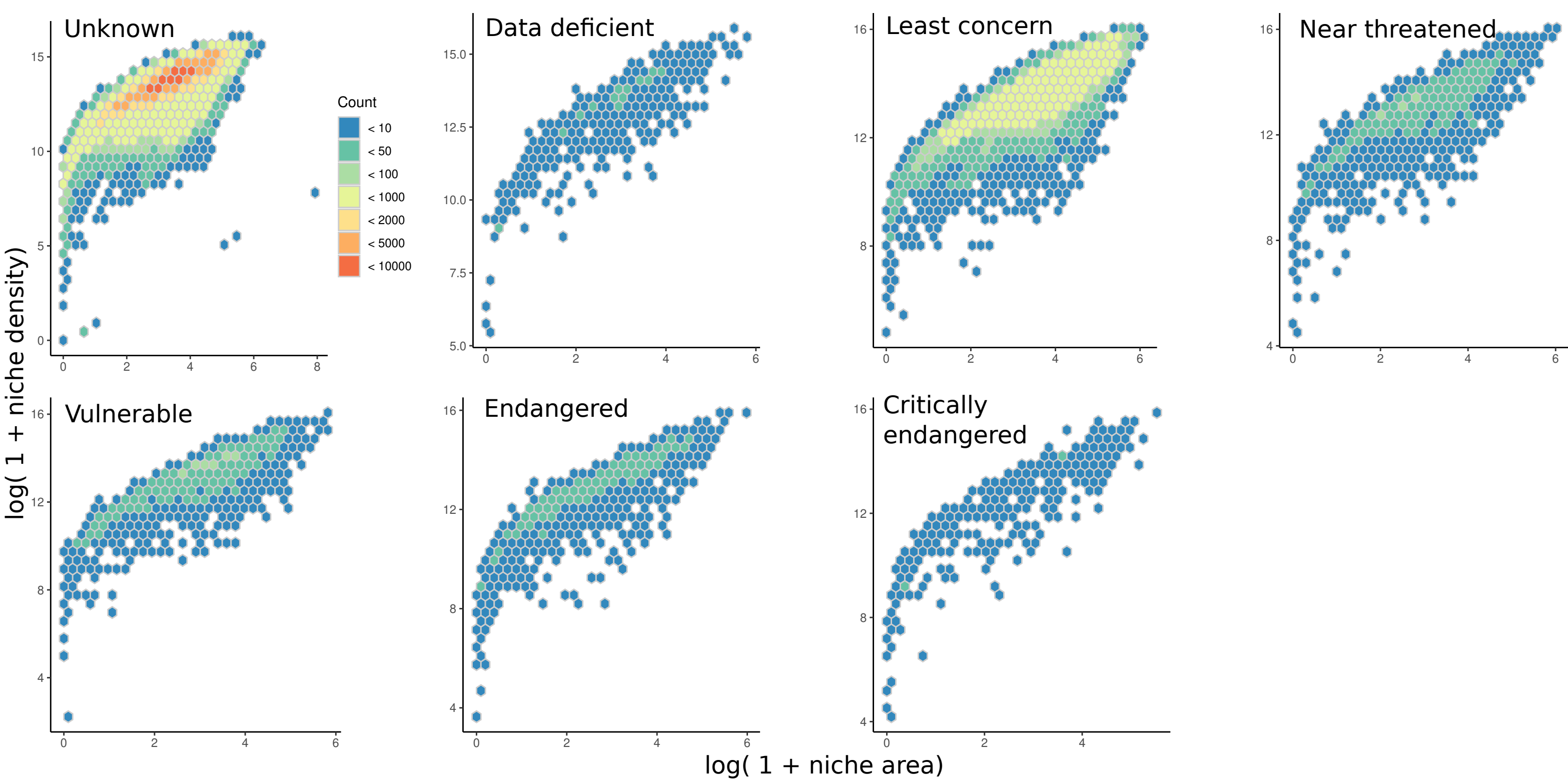

Supplement: Supplementary file 1 — Figure S1. Niche area—defined as the area of the minimum convex polygon in niche space—was positively related to niche density, which we defined as the sum of the geographic cells which contain environmental conditions within the minimum convex polygon that is the species niche. Figure S2. Constraining the species considered and environmental niche space to only the Americas resulted in findings qualitatively similar to the main text. Figure S3. Given the set of null species simulations, we see a weak positive relationship between geographic range size and niche area. Figure S4. Given the set of null species simulations, we see a weak positive relationship between geographic range size and niche density. Figure S5. Geographic range size estimation using minimum convex polygons (x‐axis) compared to estimates from alpha hulls across a range of parameterizations of α. Figure S6. Correlations between geographic range size estimates (right) and niche density estimates (left) at different levels of data thresholding (either 5% or 10% extreme points removed from the geographic range). Figure S7. The relationship between geographic range size and climatic niche density was not strongly affected by the removal of extreme geographic values prior to estimation of geographic range size and climatic niche density for the 500 randomly sampled species explored. Figure S8. Niche area – defined as the area of the minimum convex polygon in niche space – was positively related to niche density, which we defined as the sum of the geographic cells which contain environmental conditions within the minimum convex polygon that is the species niche. Figure S9. Geographic range size was positively related to niche density, regardless of IUCN threat status. Figure S10. The fraction of records per species considered in our analyses which came from iNaturalist observations. Table S1. Pearson's correlations between both geographic range size (as estimated using minimum convex polygon) and niche [file JANE-94-1221-s001.zip › iucnNiche.pdf]

log( 1 + niche density)

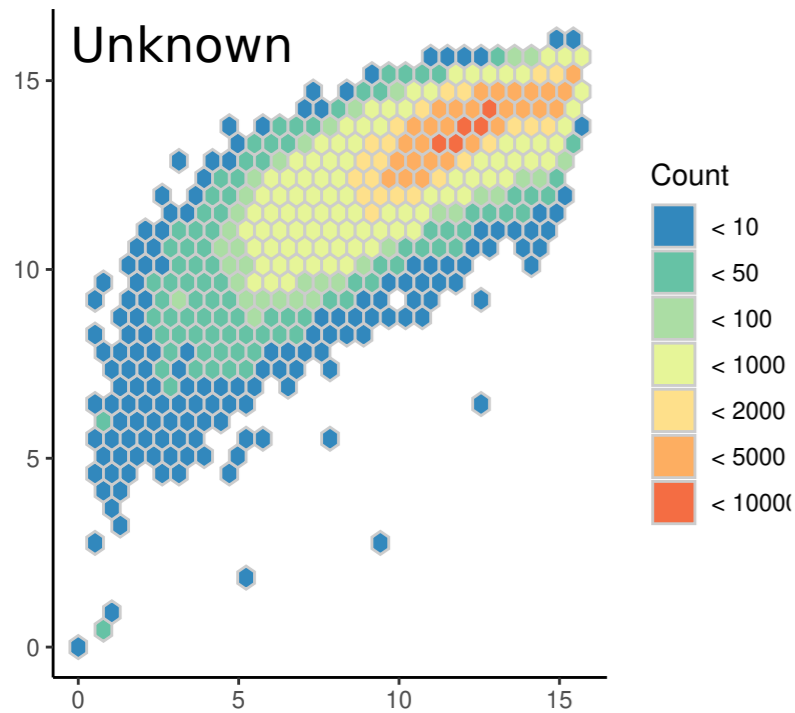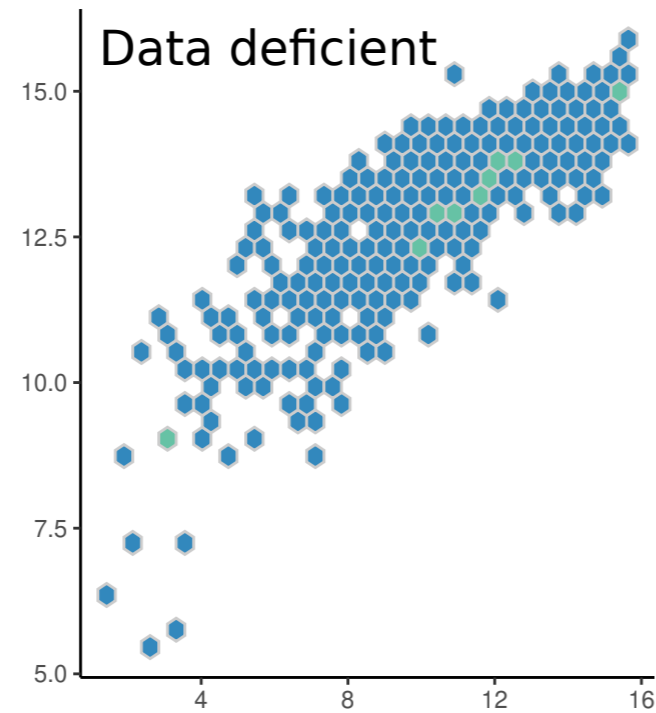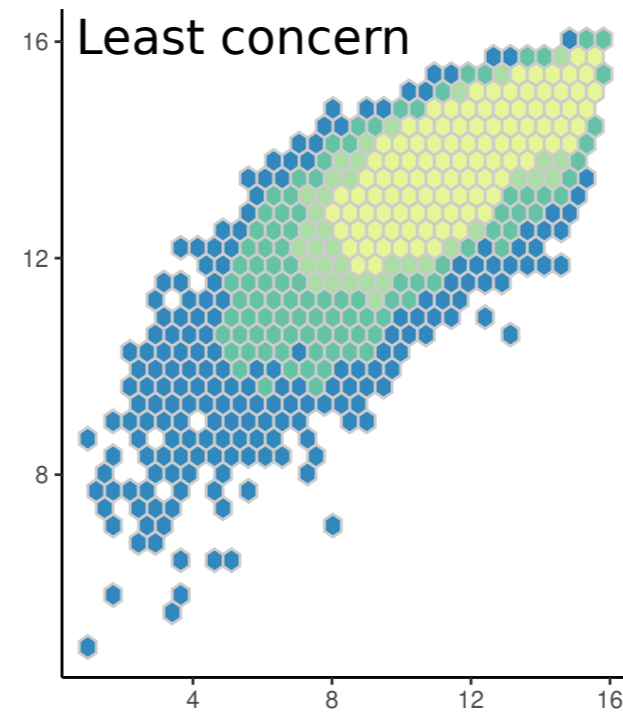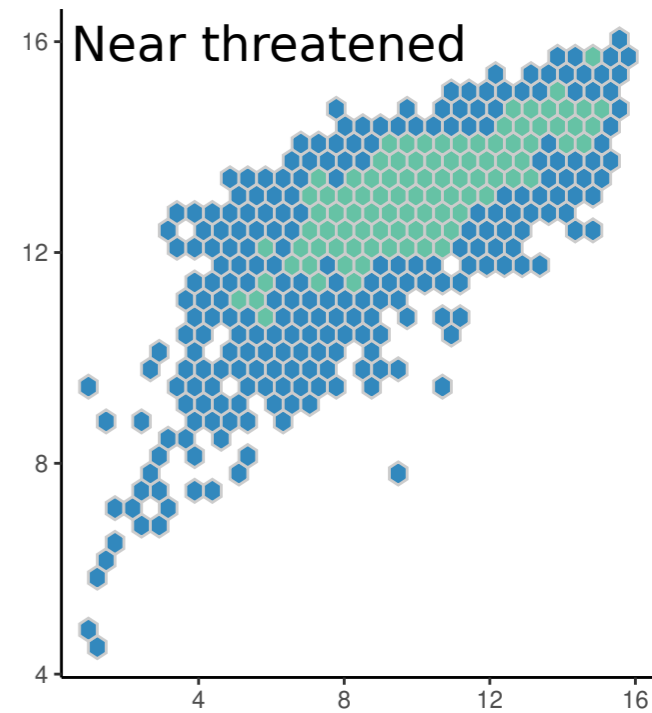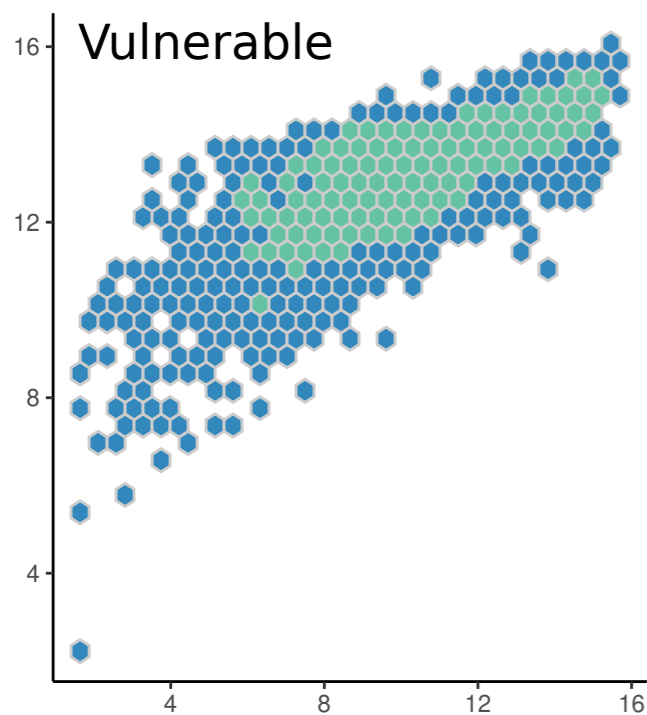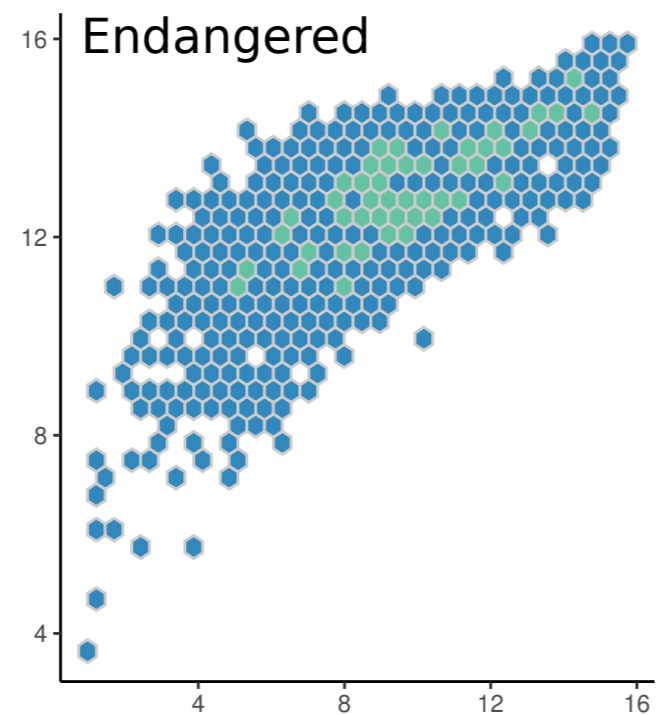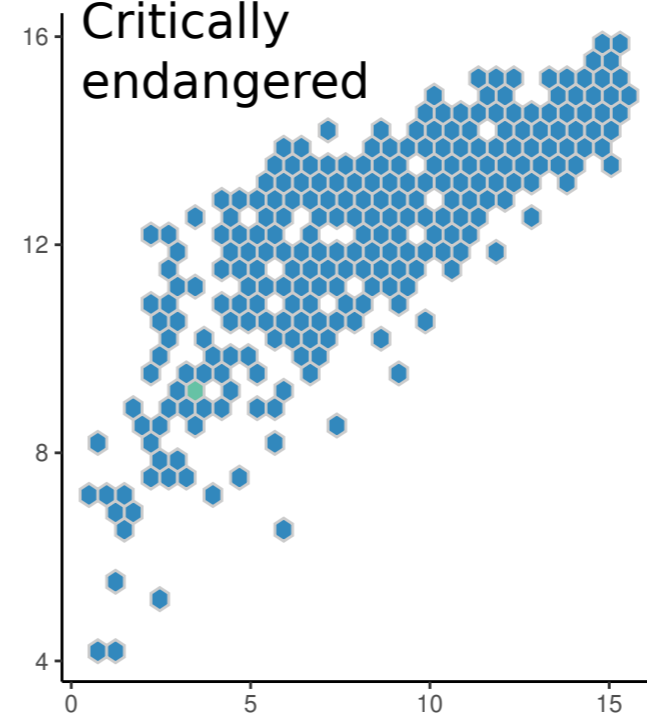

log( 1 + geographic range size)

Supplement: Supplementary file 1 — Figure S1. Niche area—defined as the area of the minimum convex polygon in niche space—was positively related to niche density, which we defined as the sum of the geographic cells which contain environmental conditions within the minimum convex polygon that is the species niche. Figure S2. Constraining the species considered and environmental niche space to only the Americas resulted in findings qualitatively similar to the main text. Figure S3. Given the set of null species simulations, we see a weak positive relationship between geographic range size and niche area. Figure S4. Given the set of null species simulations, we see a weak positive relationship between geographic range size and niche density. Figure S5. Geographic range size estimation using minimum convex polygons (x‐axis) compared to estimates from alpha hulls across a range of parameterizations of α. Figure S6. Correlations between geographic range size estimates (right) and niche density estimates (left) at different levels of data thresholding (either 5% or 10% extreme points removed from the geographic range). Figure S7. The relationship between geographic range size and climatic niche density was not strongly affected by the removal of extreme geographic values prior to estimation of geographic range size and climatic niche density for the 500 randomly sampled species explored. Figure S8. Niche area – defined as the area of the minimum convex polygon in niche space – was positively related to niche density, which we defined as the sum of the geographic cells which contain environmental conditions within the minimum convex polygon that is the species niche. Figure S9. Geographic range size was positively related to niche density, regardless of IUCN threat status. Figure S10. The fraction of records per species considered in our analyses which came from iNaturalist observations. Table S1. Pearson's correlations between both geographic range size (as estimated using minimum convex polygon) and niche [file JANE-94-1221-s001.zip › iucnNicheGeog.pdf]

## Niche density

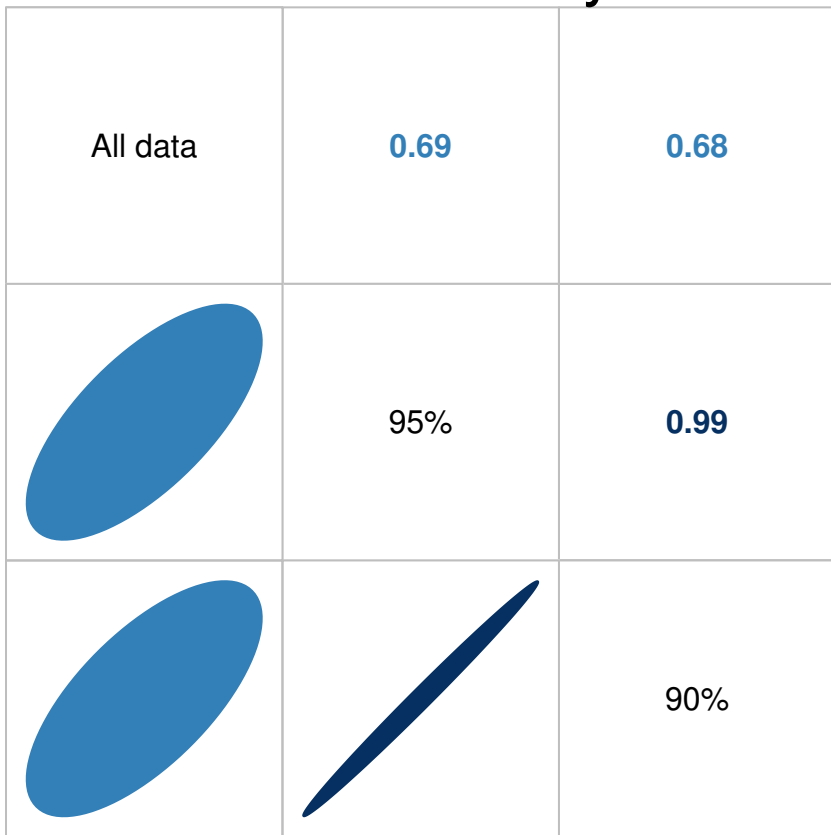

## Geographic range size

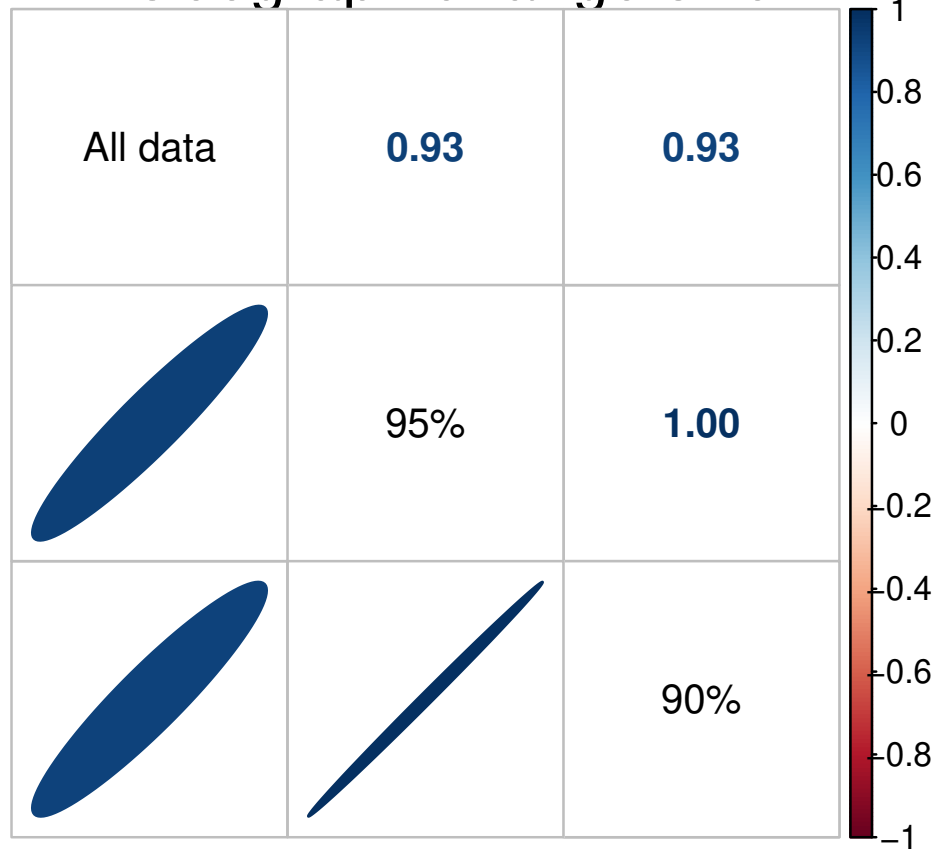

Supplement: Supplementary file 1 — Figure S1. Niche area—defined as the area of the minimum convex polygon in niche space—was positively related to niche density, which we defined as the sum of the geographic cells which contain environmental conditions within the minimum convex polygon that is the species niche. Figure S2. Constraining the species considered and environmental niche space to only the Americas resulted in findings qualitatively similar to the main text. Figure S3. Given the set of null species simulations, we see a weak positive relationship between geographic range size and niche area. Figure S4. Given the set of null species simulations, we see a weak positive relationship between geographic range size and niche density. Figure S5. Geographic range size estimation using minimum convex polygons (x‐axis) compared to estimates from alpha hulls across a range of parameterizations of α. Figure S6. Correlations between geographic range size estimates (right) and niche density estimates (left) at different levels of data thresholding (either 5% or 10% extreme points removed from the geographic range). Figure S7. The relationship between geographic range size and climatic niche density was not strongly affected by the removal of extreme geographic values prior to estimation of geographic range size and climatic niche density for the 500 randomly sampled species explored. Figure S8. Niche area – defined as the area of the minimum convex polygon in niche space – was positively related to niche density, which we defined as the sum of the geographic cells which contain environmental conditions within the minimum convex polygon that is the species niche. Figure S9. Geographic range size was positively related to niche density, regardless of IUCN threat status. Figure S10. The fraction of records per species considered in our analyses which came from iNaturalist observations. Table S1. Pearson's correlations between both geographic range size (as estimated using minimum convex polygon) and niche [file JANE-94-1221-s001.zip › ngCutCors.pdf]

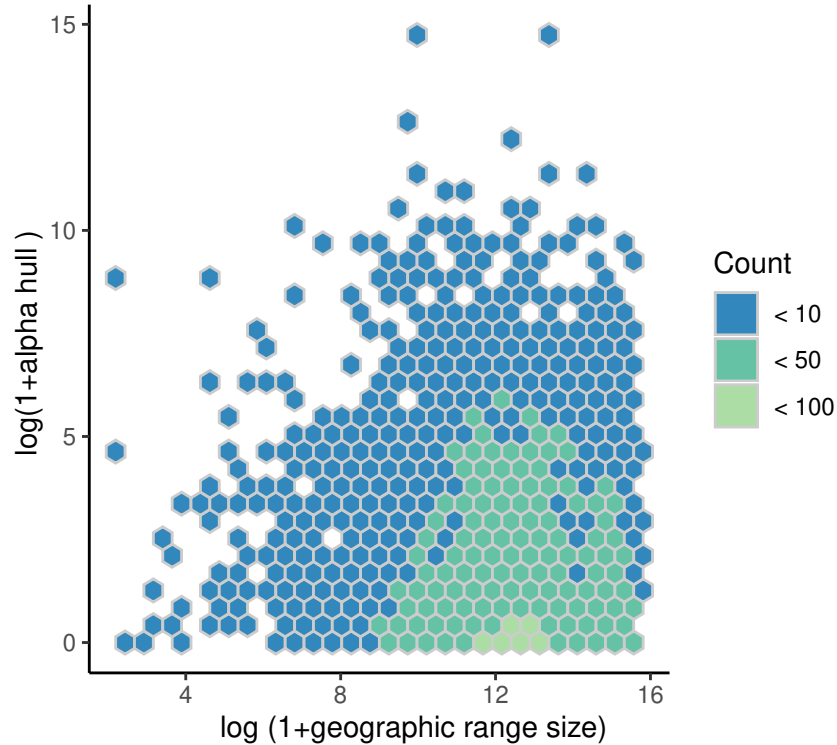

Supplement: Supplementary file 1 — Figure S1. Niche area—defined as the area of the minimum convex polygon in niche space—was positively related to niche density, which we defined as the sum of the geographic cells which contain environmental conditions within the minimum convex polygon that is the species niche. Figure S2. Constraining the species considered and environmental niche space to only the Americas resulted in findings qualitatively similar to the main text. Figure S3. Given the set of null species simulations, we see a weak positive relationship between geographic range size and niche area. Figure S4. Given the set of null species simulations, we see a weak positive relationship between geographic range size and niche density. Figure S5. Geographic range size estimation using minimum convex polygons (x‐axis) compared to estimates from alpha hulls across a range of parameterizations of α. Figure S6. Correlations between geographic range size estimates (right) and niche density estimates (left) at different levels of data thresholding (either 5% or 10% extreme points removed from the geographic range). Figure S7. The relationship between geographic range size and climatic niche density was not strongly affected by the removal of extreme geographic values prior to estimation of geographic range size and climatic niche density for the 500 randomly sampled species explored. Figure S8. Niche area – defined as the area of the minimum convex polygon in niche space – was positively related to niche density, which we defined as the sum of the geographic cells which contain environmental conditions within the minimum convex polygon that is the species niche. Figure S9. Geographic range size was positively related to niche density, regardless of IUCN threat status. Figure S10. The fraction of records per species considered in our analyses which came from iNaturalist observations. Table S1. Pearson's correlations between both geographic range size (as estimated using minimum convex polygon) and niche [file JANE-94-1221-s001.zip › nicheAlphaHex1.pdf]

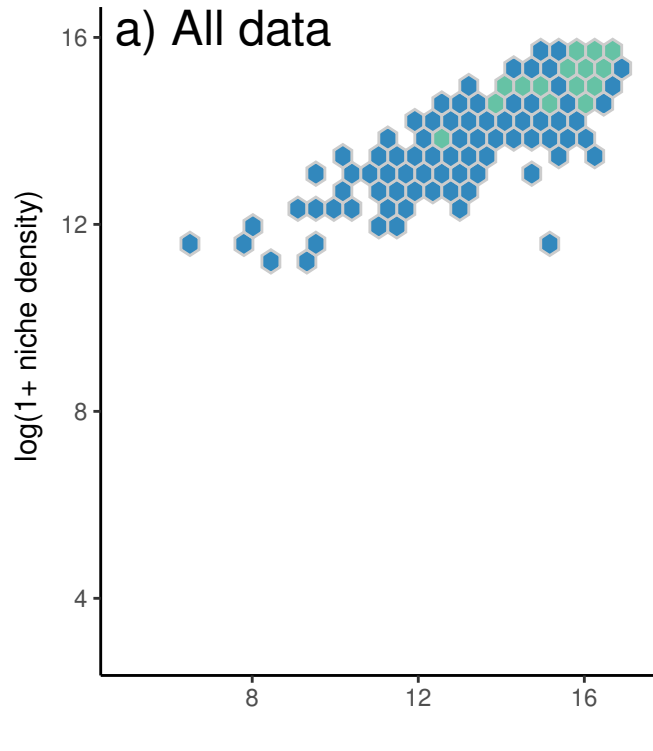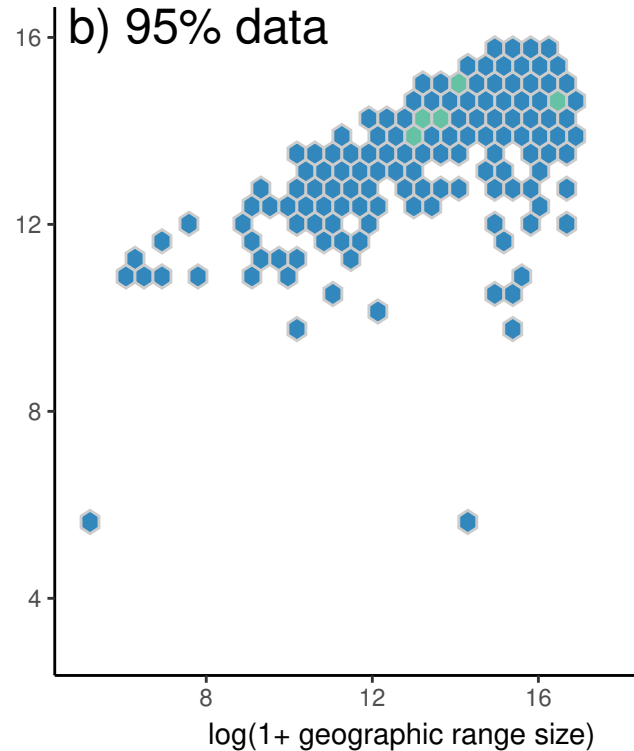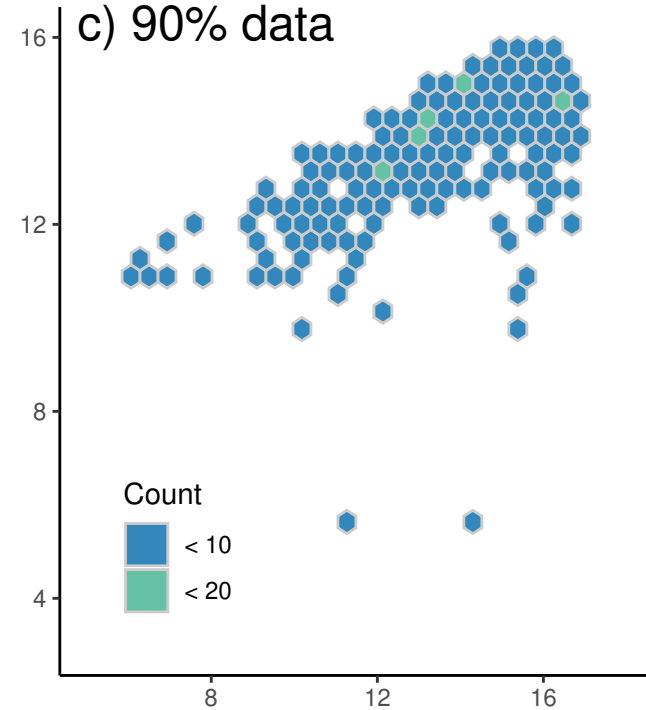

Supplement: Supplementary file 1 — Figure S1. Niche area—defined as the area of the minimum convex polygon in niche space—was positively related to niche density, which we defined as the sum of the geographic cells which contain environmental conditions within the minimum convex polygon that is the species niche. Figure S2. Constraining the species considered and environmental niche space to only the Americas resulted in findings qualitatively similar to the main text. Figure S3. Given the set of null species simulations, we see a weak positive relationship between geographic range size and niche area. Figure S4. Given the set of null species simulations, we see a weak positive relationship between geographic range size and niche density. Figure S5. Geographic range size estimation using minimum convex polygons (x‐axis) compared to estimates from alpha hulls across a range of parameterizations of α. Figure S6. Correlations between geographic range size estimates (right) and niche density estimates (left) at different levels of data thresholding (either 5% or 10% extreme points removed from the geographic range). Figure S7. The relationship between geographic range size and climatic niche density was not strongly affected by the removal of extreme geographic values prior to estimation of geographic range size and climatic niche density for the 500 randomly sampled species explored. Figure S8. Niche area – defined as the area of the minimum convex polygon in niche space – was positively related to niche density, which we defined as the sum of the geographic cells which contain environmental conditions within the minimum convex polygon that is the species niche. Figure S9. Geographic range size was positively related to niche density, regardless of IUCN threat status. Figure S10. The fraction of records per species considered in our analyses which came from iNaturalist observations. Table S1. Pearson's correlations between both geographic range size (as estimated using minimum convex polygon) and niche [file JANE-94-1221-s001.zip › nicheGeogHexCut0.pdf]

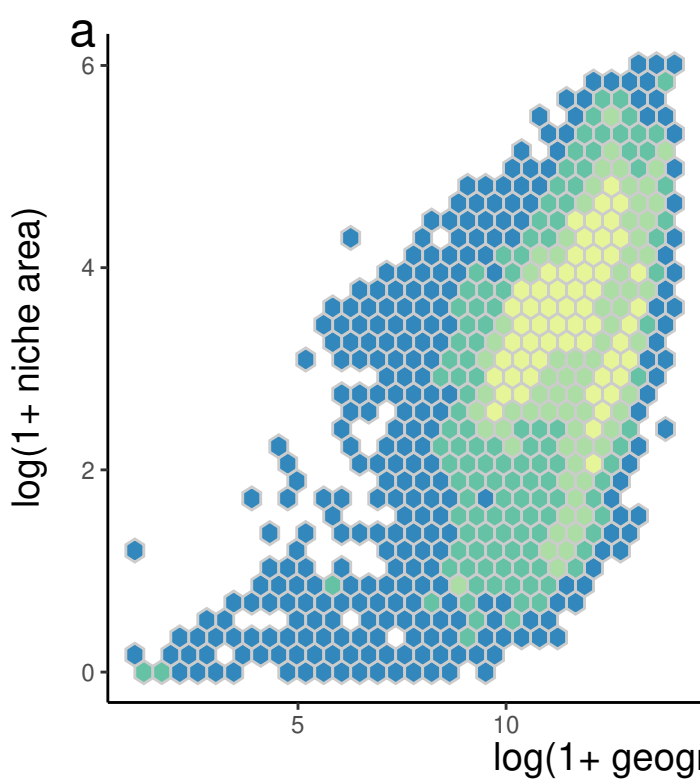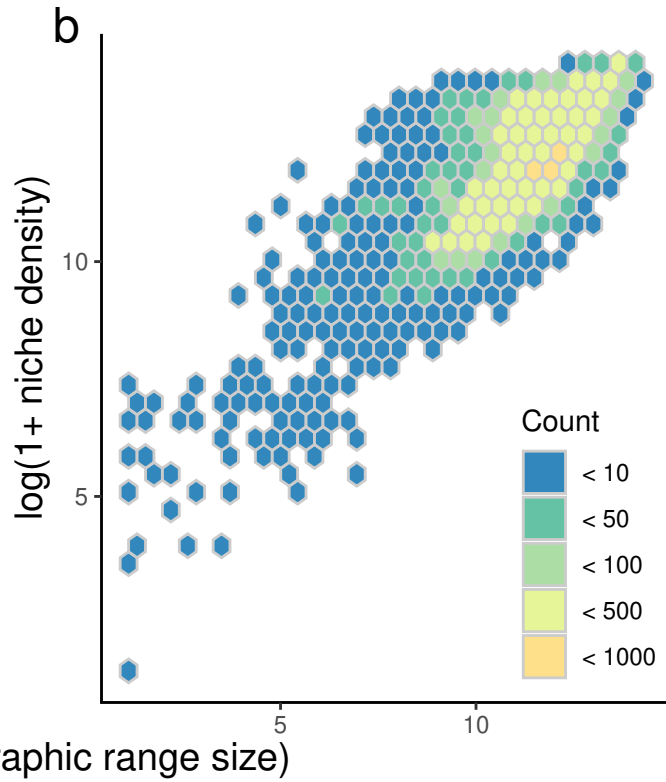

Supplement: Supplementary file 1 — Figure S1. Niche area—defined as the area of the minimum convex polygon in niche space—was positively related to niche density, which we defined as the sum of the geographic cells which contain environmental conditions within the minimum convex polygon that is the species niche. Figure S2. Constraining the species considered and environmental niche space to only the Americas resulted in findings qualitatively similar to the main text. Figure S3. Given the set of null species simulations, we see a weak positive relationship between geographic range size and niche area. Figure S4. Given the set of null species simulations, we see a weak positive relationship between geographic range size and niche density. Figure S5. Geographic range size estimation using minimum convex polygons (x‐axis) compared to estimates from alpha hulls across a range of parameterizations of α. Figure S6. Correlations between geographic range size estimates (right) and niche density estimates (left) at different levels of data thresholding (either 5% or 10% extreme points removed from the geographic range). Figure S7. The relationship between geographic range size and climatic niche density was not strongly affected by the removal of extreme geographic values prior to estimation of geographic range size and climatic niche density for the 500 randomly sampled species explored. Figure S8. Niche area – defined as the area of the minimum convex polygon in niche space – was positively related to niche density, which we defined as the sum of the geographic cells which contain environmental conditions within the minimum convex polygon that is the species niche. Figure S9. Geographic range size was positively related to niche density, regardless of IUCN threat status. Figure S10. The fraction of records per species considered in our analyses which came from iNaturalist observations. Table S1. Pearson's correlations between both geographic range size (as estimated using minimum convex polygon) and niche [file JANE-94-1221-s001.zip › nicheGeogHexNA.pdf]

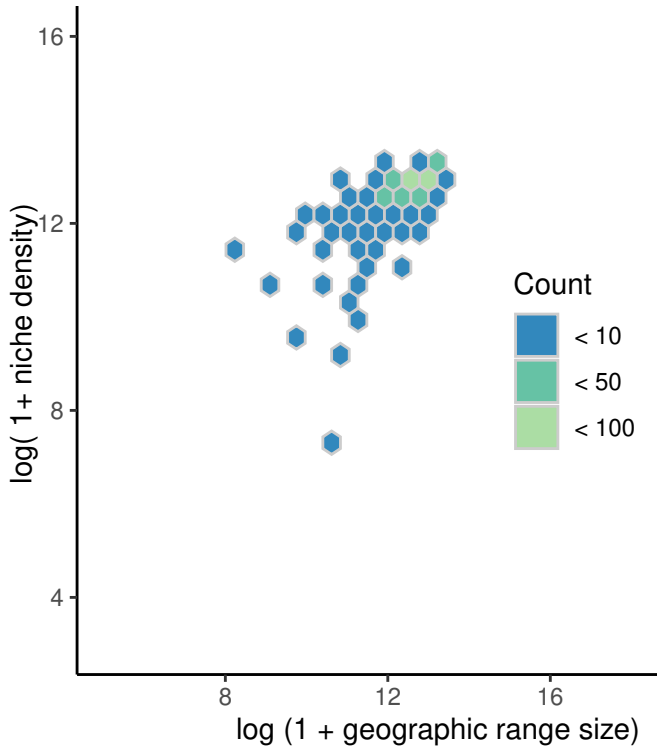

Supplement: Supplementary file 1 — Figure S1. Niche area—defined as the area of the minimum convex polygon in niche space—was positively related to niche density, which we defined as the sum of the geographic cells which contain environmental conditions within the minimum convex polygon that is the species niche. Figure S2. Constraining the species considered and environmental niche space to only the Americas resulted in findings qualitatively similar to the main text. Figure S3. Given the set of null species simulations, we see a weak positive relationship between geographic range size and niche area. Figure S4. Given the set of null species simulations, we see a weak positive relationship between geographic range size and niche density. Figure S5. Geographic range size estimation using minimum convex polygons (x‐axis) compared to estimates from alpha hulls across a range of parameterizations of α. Figure S6. Correlations between geographic range size estimates (right) and niche density estimates (left) at different levels of data thresholding (either 5% or 10% extreme points removed from the geographic range). Figure S7. The relationship between geographic range size and climatic niche density was not strongly affected by the removal of extreme geographic values prior to estimation of geographic range size and climatic niche density for the 500 randomly sampled species explored. Figure S8. Niche area – defined as the area of the minimum convex polygon in niche space – was positively related to niche density, which we defined as the sum of the geographic cells which contain environmental conditions within the minimum convex polygon that is the species niche. Figure S9. Geographic range size was positively related to niche density, regardless of IUCN threat status. Figure S10. The fraction of records per species considered in our analyses which came from iNaturalist observations. Table S1. Pearson's correlations between both geographic range size (as estimated using minimum convex polygon) and niche [file JANE-94-1221-s001.zip › nicheGeogHexUSA2.pdf]

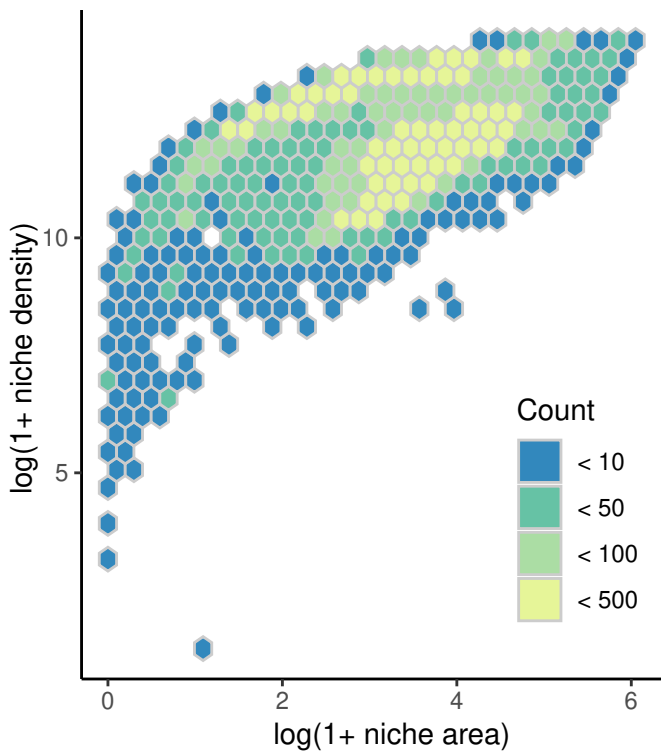

Supplement: Supplementary file 1 — Figure S1. Niche area—defined as the area of the minimum convex polygon in niche space—was positively related to niche density, which we defined as the sum of the geographic cells which contain environmental conditions within the minimum convex polygon that is the species niche. Figure S2. Constraining the species considered and environmental niche space to only the Americas resulted in findings qualitatively similar to the main text. Figure S3. Given the set of null species simulations, we see a weak positive relationship between geographic range size and niche area. Figure S4. Given the set of null species simulations, we see a weak positive relationship between geographic range size and niche density. Figure S5. Geographic range size estimation using minimum convex polygons (x‐axis) compared to estimates from alpha hulls across a range of parameterizations of α. Figure S6. Correlations between geographic range size estimates (right) and niche density estimates (left) at different levels of data thresholding (either 5% or 10% extreme points removed from the geographic range). Figure S7. The relationship between geographic range size and climatic niche density was not strongly affected by the removal of extreme geographic values prior to estimation of geographic range size and climatic niche density for the 500 randomly sampled species explored. Figure S8. Niche area – defined as the area of the minimum convex polygon in niche space – was positively related to niche density, which we defined as the sum of the geographic cells which contain environmental conditions within the minimum convex polygon that is the species niche. Figure S9. Geographic range size was positively related to niche density, regardless of IUCN threat status. Figure S10. The fraction of records per species considered in our analyses which came from iNaturalist observations. Table S1. Pearson's correlations between both geographic range size (as estimated using minimum convex polygon) and niche [file JANE-94-1221-s001.zip › nicheHexNA.pdf]

Count ■ < 10 ■ < 50 ■ < 100 ■ < 1000 ■ < 2000

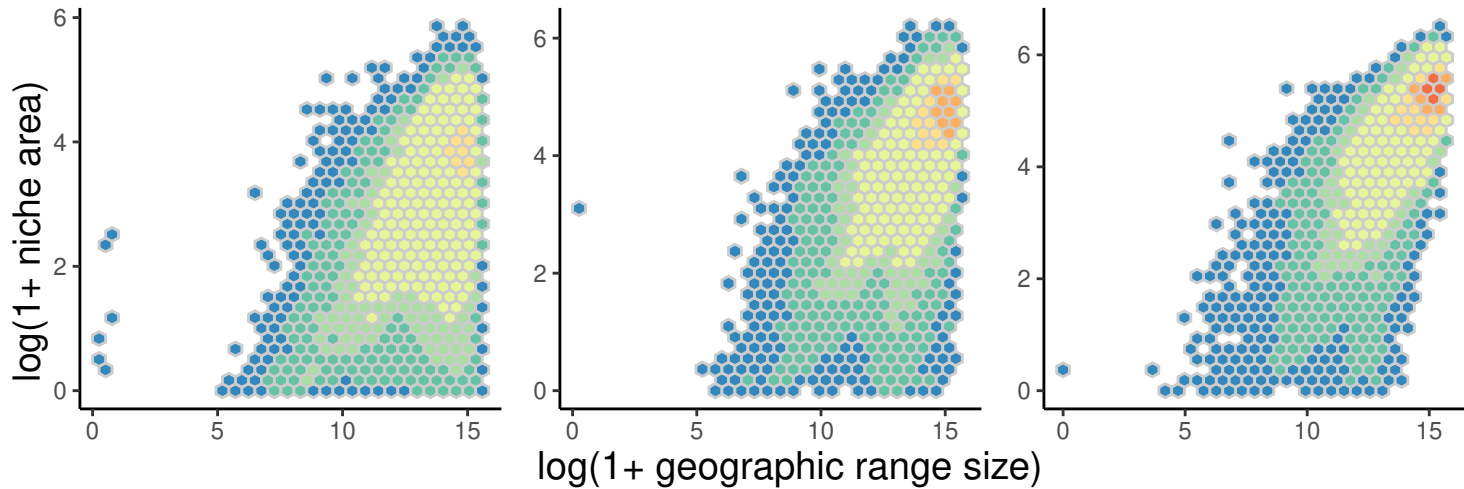

Supplement: Supplementary file 1 — Figure S1. Niche area—defined as the area of the minimum convex polygon in niche space—was positively related to niche density, which we defined as the sum of the geographic cells which contain environmental conditions within the minimum convex polygon that is the species niche. Figure S2. Constraining the species considered and environmental niche space to only the Americas resulted in findings qualitatively similar to the main text. Figure S3. Given the set of null species simulations, we see a weak positive relationship between geographic range size and niche area. Figure S4. Given the set of null species simulations, we see a weak positive relationship between geographic range size and niche density. Figure S5. Geographic range size estimation using minimum convex polygons (x‐axis) compared to estimates from alpha hulls across a range of parameterizations of α. Figure S6. Correlations between geographic range size estimates (right) and niche density estimates (left) at different levels of data thresholding (either 5% or 10% extreme points removed from the geographic range). Figure S7. The relationship between geographic range size and climatic niche density was not strongly affected by the removal of extreme geographic values prior to estimation of geographic range size and climatic niche density for the 500 randomly sampled species explored. Figure S8. Niche area – defined as the area of the minimum convex polygon in niche space – was positively related to niche density, which we defined as the sum of the geographic cells which contain environmental conditions within the minimum convex polygon that is the species niche. Figure S9. Geographic range size was positively related to niche density, regardless of IUCN threat status. Figure S10. The fraction of records per species considered in our analyses which came from iNaturalist observations. Table S1. Pearson's correlations between both geographic range size (as estimated using minimum convex polygon) and niche [file JANE-94-1221-s001.zip › nullHexArea.pdf]

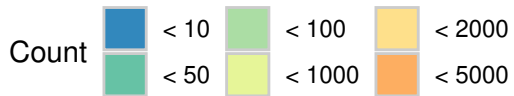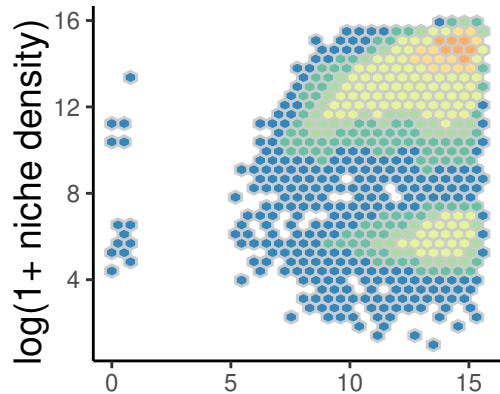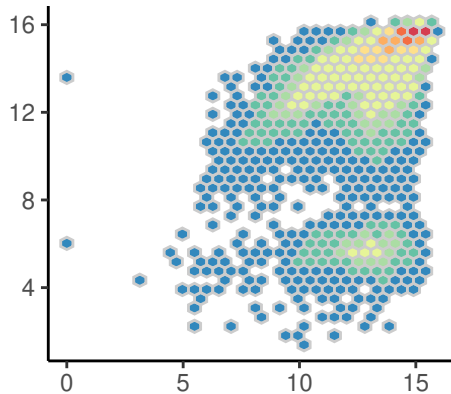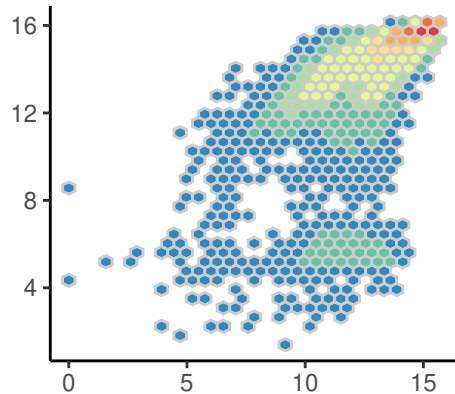

$\log(1 + \text{geographic range size})$

Supplement: Supplementary file 1 — Figure S1. Niche area—defined as the area of the minimum convex polygon in niche space—was positively related to niche density, which we defined as the sum of the geographic cells which contain environmental conditions within the minimum convex polygon that is the species niche. Figure S2. Constraining the species considered and environmental niche space to only the Americas resulted in findings qualitatively similar to the main text. Figure S3. Given the set of null species simulations, we see a weak positive relationship between geographic range size and niche area. Figure S4. Given the set of null species simulations, we see a weak positive relationship between geographic range size and niche density. Figure S5. Geographic range size estimation using minimum convex polygons (x‐axis) compared to estimates from alpha hulls across a range of parameterizations of α. Figure S6. Correlations between geographic range size estimates (right) and niche density estimates (left) at different levels of data thresholding (either 5% or 10% extreme points removed from the geographic range). Figure S7. The relationship between geographic range size and climatic niche density was not strongly affected by the removal of extreme geographic values prior to estimation of geographic range size and climatic niche density for the 500 randomly sampled species explored. Figure S8. Niche area – defined as the area of the minimum convex polygon in niche space – was positively related to niche density, which we defined as the sum of the geographic cells which contain environmental conditions within the minimum convex polygon that is the species niche. Figure S9. Geographic range size was positively related to niche density, regardless of IUCN threat status. Figure S10. The fraction of records per species considered in our analyses which came from iNaturalist observations. Table S1. Pearson's correlations between both geographic range size (as estimated using minimum convex polygon) and niche [file JANE-94-1221-s001.zip › nullHexDensity.pdf]
